# PTEN-L is a novel protein phosphatase for ubiquitin dephosphorylation to inhibit PINK1–Parkin-mediated mitophagy

**DOI:** 10.1038/s41422-018-0056-0

**Published:** 2018-06-22

**Authors:** Liming Wang, Yik-Lam Cho, Yancheng Tang, Jigang Wang, Jung-Eun Park, Yajun Wu, Chunxin Wang, Yan Tong, Ritu Chawla, Jianbin Zhang, Yin Shi, Shuo Deng, Guang Lu, Yihua Wu, Hayden Weng-Siong Tan, Pornteera Pawijit, Grace Gui-Yin Lim, Hui-Ying Chan, Jingzi Zhang, Lei Fang, Hanry Yu, Yih-Cherng Liou, Mallilankaraman Karthik, Boon-Huat Bay, Kah-Leong Lim, Siu-Kwan Sze, Celestial T. Yap, Han-Ming Shen

**Affiliations:** 1 Department of Physiology, Yong Loo Lin School of Medicine, National University of Singapore, Singapore, Singapore; 2 School of Chinese Medicine, Hong Kong Baptist University, Kowloon Tong, Hong Kong China; 3 School of Biological Sciences, Nanyang Technological University, Singapore, Singapore; 4 Department of Anatomy, National University of Singapore, Singapore, Singapore; 5 Biochemistry Section, Surgical Neurology Branch, National Institute of Neurological Disorders and Stroke, National Institutes of Health, Bethesda, MD 20892 USA; 6 Department of Biological Sciences, Faculty of Science, National University of Singapore, Singapore, Singapore; 7 Department of Oncology, Clinical Research Institute, Zhejiang Provincial People’s Hospital, People’s Hospital of Hangzhou Medical College, Hangzhou, Zhejiang China; 8 Department of Toxicology, Zhejiang University School of Public Health, Hangzhou, Zhejiang China; 9 Graduate School for Integrative Sciences and Engineering, National University of Singapore, Singapore, Singapore; 10 National Neuroscience Institute, Singapore, Singapore; 11 Jiangsu Key Laboratory of Molecular Medicine, Model Animal Research Center, Medical School of Nanjing University, Nanjing, Jiangsu China; 12 Mechanobiology Institute, National University of Singapore, Singapore, Singapore; 13 Institute of Bioengineering and Nanotechnology, A*STAR, Singapore, Singapore

## Abstract

Mitophagy is an important type of selective autophagy for specific elimination of damaged mitochondria. PTEN-induced putative kinase protein 1 (PINK1)-catalyzed phosphorylation of ubiquitin (Ub) plays a critical role in the onset of PINK1–Parkin-mediated mitophagy. Phosphatase and tensin homolog (PTEN)-long (PTEN-L) is a newly identified isoform of PTEN, with addition of 173 amino acids to its N-terminus. Here we report that PTEN-L is a novel negative regulator of mitophagy via its protein phosphatase activity against phosphorylated ubiquitin. We found that PTEN-L localizes at the outer mitochondrial membrane (OMM) and overexpression of PTEN-L inhibits, whereas deletion of *PTEN-L* promotes, mitophagy induced by various mitochondria-damaging agents. Mechanistically, PTEN-L is capable of effectively preventing Parkin mitochondrial translocation, reducing Parkin phosphorylation, maintaining its closed inactive conformation, and inhibiting its E3 ligase activity. More importantly, PTEN-L reduces the level of phosphorylated ubiquitin (pSer65-Ub) in vivo, and in vitro phosphatase assay confirms that PTEN-L dephosphorylates pSer65-Ub via its protein phosphatase activity, independently of its lipid phosphatase function. Taken together, our findings demonstrate a novel function of PTEN-L as a protein phosphatase for ubiquitin, which counteracts PINK1-mediated ubiquitin phosphorylation leading to blockage of the feedforward mechanisms in mitophagy induction and eventual suppression of mitophagy. Thus, understanding this novel function of PTEN-L provides a key missing piece in the molecular puzzle controlling mitophagy, a critical process in many important human diseases including neurodegenerative disorders such as Parkinson’s disease.

## Introduction

Mitophagy is a selective form of autophagy for elimination of damaged mitochondria.^1^ Defective mitophagy is well known to be implicated in the pathogenesis of neurodegenerative disorders, in particular Parkinson’s disease.^2,3^ The E3 ubiquitin (ligase Parkin (encoded by the *PARK2* gene) and the serine/threonine kinase PTEN-induced putative kinase 1 (PINK1; encoded by the *PINK1/PARK6* gene) are the two key molecules in the regulation of mitophagy.^4–7^ Once mitochondria are damaged upon depolarization of mitochondrial membrane, PINK1 is stabilized on the outer mitochondrial membrane (OMM)^8,9^ and phosphorylates ubiquitin (Ub)^10–12^ and Parkin.^13–15^ Importantly, phosphorylated ubiquitin (pSer65-Ub), especially the pSer65-Ub chains, functions as a potent Parkin activator and Parkin receptor, leading to enhanced ubiquitination of proteins at the OMM and onset of mitophagy.^16–19^ Therefore, PINK1-mediated phosphorylation and Parkin-mediated ubiquitination form a feedforward mechanism of mitophagy.^4, 20^ At present, the negative regulatory mechanisms against Parkin-mediated ubiquitination have been reported, with the discovery of several deubiquitinases including USP8, USP15 and USP30 that are able to cause the deubiquitination of Parkin and/or OMM proteins.^21–24^ Interestingly, an earlier nuclear magnetic resonance (NMR) study suggested that ubiquitin structure was altered upon phosphorylation at Ser65 by PINK1 that makes pSer65-Ub resistant to deubiquitinases.^25^ Therefore, it is believed that the dephosphorylation of ubiquitin is a more critical negative regulatory mechanism in the regulation of mitophagy and the presence of specific protein phosphatases against PINK1-mediated phosphorylation has been speculated.^4,26^ However, the mechanisms for negative regulation of the PINK1-mediated ubiquitin and Parkin phosphorylation remain elusive up to date.

Phosphatase and tensin homolog (PTEN) is an important tumor suppressor with both lipid phosphatase and protein phosphatase activities.^27–29^ One of the canonical functions of PTEN is to inhibit the phosphatidylinositol 3-kinase (PI3K)–AKT pathway through its lipid phosphatase activity^30,31 ^— PTEN dephosphorylates phosphatidylinositol (3,4,5)-trisphosphate (PI(3,4,5)P3) to form phosphatidylinositol 4,5-bisphosphate (PI(4,5)P2), inhibiting AKT and its downstream signaling pathways. Recently, PTEN-Long/PTEN-L or PTENα (referred to as PTEN-L hereafter), a translational variant of PTEN, was identified as a new isoform of PTEN.^32–34^ It has been reported that PTEN-L was a secreted protein and could be detected in human serum and plasma.^34^ The alternatively translated region (ATR) includes a polyarginine stretch with homology to known cell-permeable peptides, which makes PTEN-L to be secreted from cells and enter other cells to inhibit PI3K signaling both in vitro and in vivo.^34^ Several other studies have also revealed the implication of the catalytic activity of PTEN-L in its tumor suppressive function.^35–37^ Importantly, Liang et al.^33^ reported that PTEN-L is a mitochondria-localized protein and is able to enhance cytochrome *c* oxidase activity and ATP production in mitochondria. However, at present, the biological functions of PTEN-L remain poorly studied and the effect of PTEN-L on mitophagy has not been reported.

In this study, we report that PTEN-L is a protein phosphatase for pSer65-Ub and dephosphorylation of pSer65-Ub leads to blockage of the feedforward mechanism and suppression of mitophagy. Understanding the novel function of PTEN-L in regulation of mitophagy not only offers new insights into the complicated mechanisms of mitophagy, but also provides potential therapeutic targets for diseases related to defective mitophagy and mitochondria dysfunction such as Parkinson’s disease.

## Results

### PTEN-L resides at the outer mitochondrial membrane

**Fig. 1:**
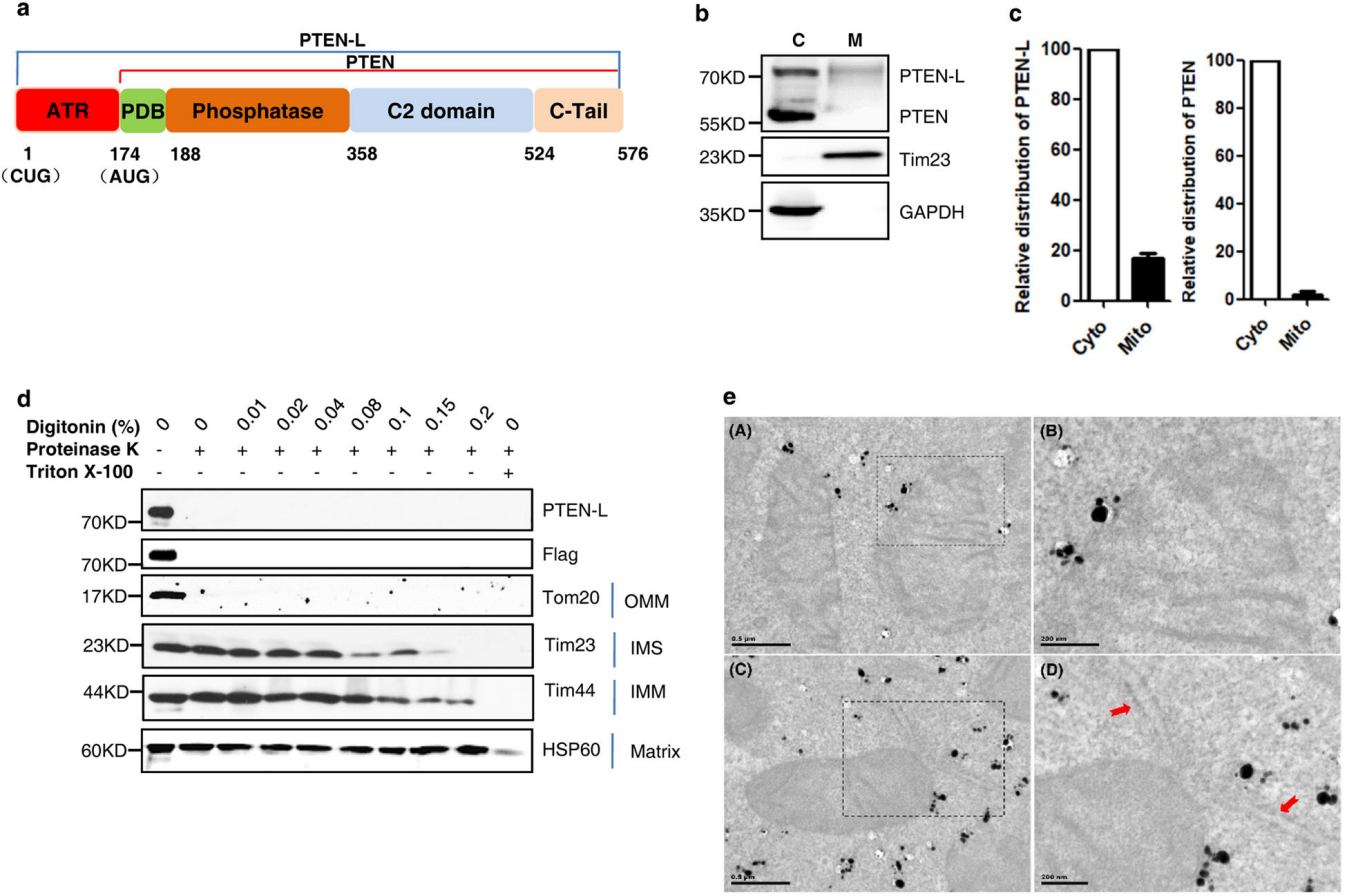
PTEN-L resides at the outer mitochondrial membrane. **a** Domain structure of PTEN-L protein. **b** Cell fractionation was performed to isolate mitochondria in HeLa cells. Tim23 and GAPDH were used as mitochondrial and cytosolic markers, respectively. C cytosol, M mitochondria. **c** Relative ratios of PTEN-L and PTEN in cytosol and mitochondria were calculated with normalization of volume. **d** Topology assay showing PTEN-L localization at the OMM. Mitochondria were isolated from YFP-Parkin-HeLa cells stably expressing Flag-PTEN-L and treated with different doses of proteinase K and digitonin. **e** Immunogold EM. YFP-Parkin-HeLa cells stably expressing Flag-PTEN-L were treated without (**A**, **B**) or with CCCP (5 µM) (**C**, **D**) for 4 h. Cells were subjected to immunoelectron microscopy with anti-Flag antibody. Panels (**B**) and (**D**) are the magnified images of boxes in (**A**) and (**C**), respectively. Arrows indicate ER structure

PTEN-L, as a novel isoform of PTEN, translates from a non-canonical CUG initiation codon, with the addition of 173 amino acids to its N-terminus^33,34^ (Fig. 1a). In this study, we aimed to investigate the regulatory effect of PTEN-L on mitophagy. We first examined the subcellular localization of the endogenous PTEN-L in HeLa cells and found that a significant amount of PTEN-L was in the mitochondrial fraction, whereas PTEN was mainly in the cytosolic fraction (Fig. 1b, c). A similar distribution pattern was observed for endogenous PTEN and PTEN-L in a variety of mouse organs/tissues (Supplementary information, Figure S1a). To study the function of PTEN-L, we established HeLa cells stably expressing Flag-PTEN-L (Supplementary information, Figure S1b) and observed a significant amount of Flag-PTEN-L in the mitochondrial fraction (Supplementary information, Figure S1c and d). Next, we performed a series of assays to examine the exact localization of PTEN-L in mitochondria. In the topology assay using purified mitochondria from the HeLa cells stably expressing PTEN-L, PTEN-L was not protected from proteinase K, similarly to Tom20, an OMM protein, and differently from Tim23, Tim44 and HSP60 that are known to localize at the outer surface of the inner membrane facing the inter-membranes space, inner surface of the mitochondrial membrane (IMM) and the mitochondrial matrix, respectively (Fig. 1d).^38^ Similar results were obtained for endogenous PTEN-L in HeLa cells (Supplementary information, Figure S1e). We further verified the colocalization of Flag-PTEN-L with Tom20 using immunohistochemistry staining with a high-resolution microscope. Consistently, a significant part of PTEN-L co-localized with Tom20 (Supplementary information, Figure S1f). Similar to the results from cell fractionation (Supplementary information, Figure S1c and d), we also observed increased PTEN-L colocalization with mitochondria in cells treated with a mitochondrial uncoupler carbonylcyanide 3-chlorophenylhydrazone (CCCP) (Supplementary information, Figure S1f and g). In contrast, no evident mitochondrial colocalization was observed for PTEN (Fig. 1b, c; Supplementary information, Figure S1h) and PTEN-L does not colocalize with calreticulin, an endoplasmic reticulum (ER) marker (Supplementary information, Figure S1i). Finally, we performed immunoelectron microscopy and found that a significant amount of gold particles attached to Flag-PTEN-L were associated with OMM (Fig. 1e), but not in mitochondrial matrix or with ER. Taken together, all our data demonstrate that a substantial amount of PTEN-L is associated with OMM.

### PTEN-L negatively regulates mitophagy induced by various mitochondria-damaging agents

**Fig. 2:**
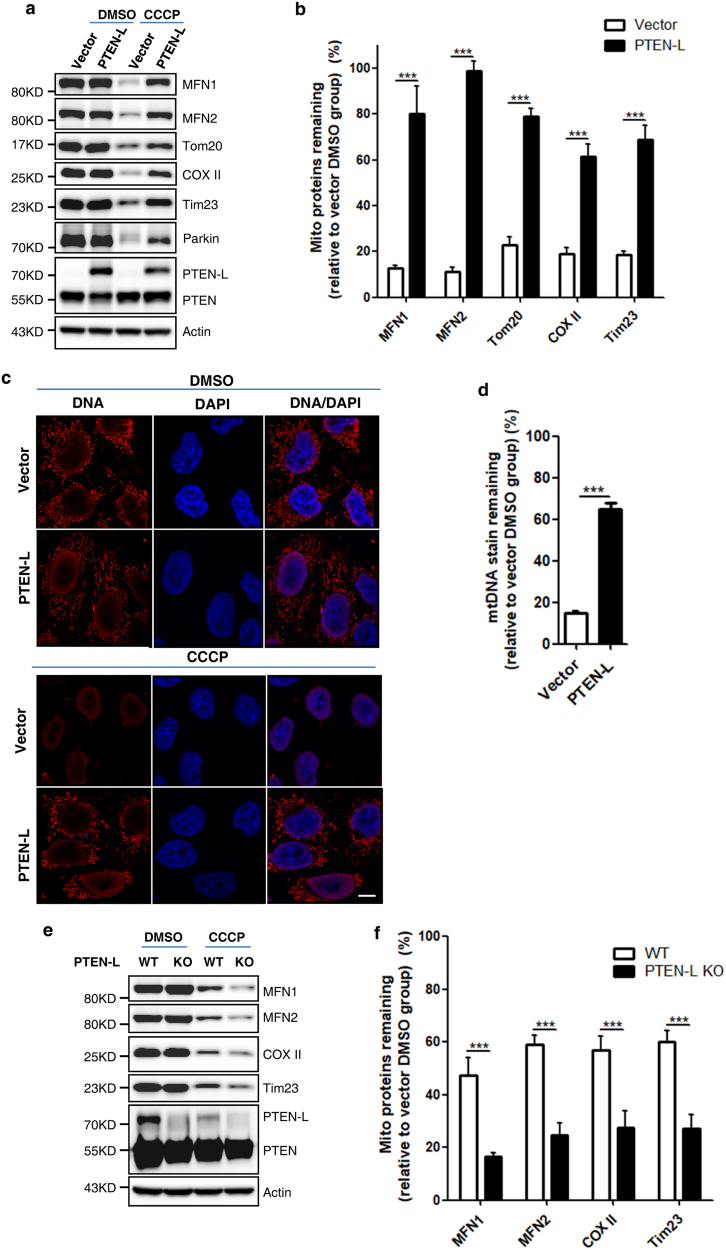
PTEN-L negatively regulates mitophagy induced by various mitochondria-damaging agents. **a** YFP-Parkin-HeLa cells with PTEN-L stable expression or control vector were treated with CCCP (5 µM) for 24 h and immunoblotting for mitochondrial proteins was performed as indicated. **b** Quantification of mitochondrial proteins from **a**. **c** Degradation of mtDNA. Representative images of YFP-Parkin-HeLa cells with PTEN-L stable expression and control vector immunostained to label mtDNA (red) after treatment with CCCP (5 µM) for 24 h. **d** Quantification of mtDNA from > 300 cells per group. Scale bar, 10 µm. **e** Wild-type (WT) and *PTEN-L* KO YFP-Parkin-HeLa cells were treated with CCCP (4 µM) for 24 h and immunoblotting was performed as indicated. **f** Quantification of data from **e**. Data in **b**, **d**, **f** are presented as mean ± SD from three independent experiments. ***P < 0.001 (two-way ANOVA, **b** and **f**), ***P < 0.0001 (Student’s *t*-test, **d**)

We attempted to study the possible regulatory effects of PTEN-L on mitophagy. First, we found that transient overexpression of PTEN-L, but not PTEN, significantly inhibited the degradation of mitochondrial proteins (Tim23 and Tom20) induced by CCCP (Supplementary information, Figure S2a and b). The inhibitory effect of PTEN-L on mitophagy was also observed by examining the degradation of mitochondria-targeted green fluorescent protein (GFP; Supplementary information, Figure S2c). Consistently, stable expression of PTEN-L markedly inhibited CCCP-induced mitophagy examined by immunoblotting, immunostaining and transmission electron microscopy (Fig. 2a, b and Supplementary information, Figure S3a, b). Since OMM proteins such as MFN1 and MFN2 could also be degraded via Parkin-mediated ubiquitination and proteasome,^39,40^ we validated the inhibitory effects of PTEN-L on mitophagy by examining changes of IMM proteins such as Tim23 and mitochondrial DNA (mtDNA)-encoded COX II.^41^ Moreover, we also examined the degradation of mtDNA nucleoids, a well-established indicator of mitophagy,^41^ and found that PTEN-L significantly blocked the degradation of mtDNA (Fig. 2c, d). Inhibition of mitophagy by PTEN-L was also observed when mitochondria were depolarized by a combination of oligomycin and antimycin A (O/A) (Supplementary information, Figure S3c and d) or valinomycin (Supplementary information, Figure S3e).

To further confirm the inhibitory effect of PTEN-L on mitophagy, we genetically knocked out PTEN-L with the CRISPR/Cas9 technique by using small guide RNAs (sgRNAs) targeting the ATR domain of PTEN-L (Supplementary information, Figure S4a, b). We confirmed that *PTEN-L* deletion did not affect PTEN expression (Supplementary information, Figure S4c) but effectively sensitized cells to mitophagy induced by CCCP (Fig. 2e, f) and O/A (Supplementary information, Figure S4d, e). We also observed that prolonged treatment with CCCP or O/A caused significant reduction of endogenous PTEN-L, similar to other mitochondrial proteins (Fig. 2e and Supplementary information, Figure S4d), indicating the possibility that mitochondria-localized endogenous PTEN-L is degraded via mitophagy.

### PTEN-L prevents Parkin mitochondrial translocation

**Fig. 3:**
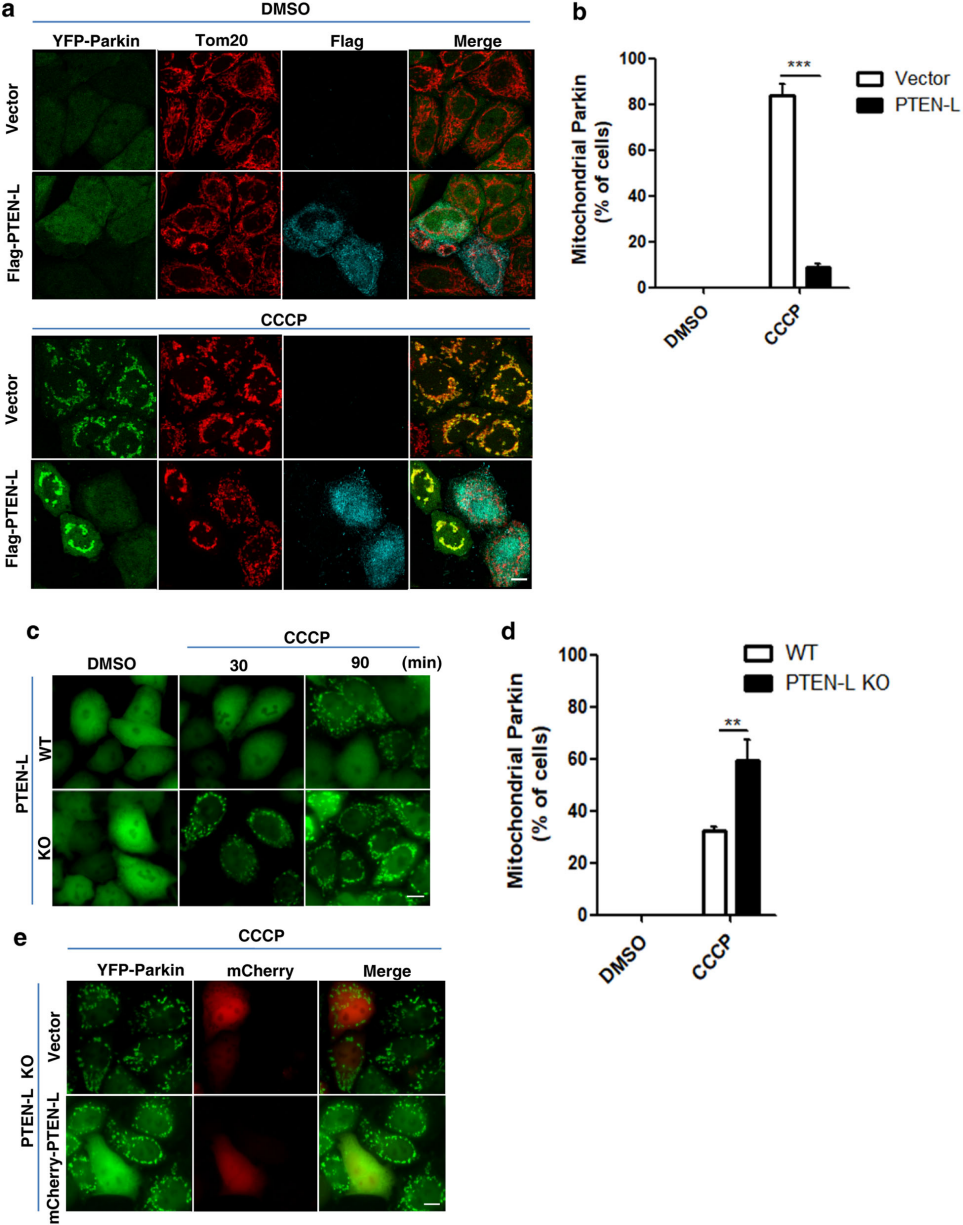
PTEN-L prevents Parkin mitochondrial translocation. **a** YFP-Parkin-HeLa cells transiently transfected with Flag-PTEN-L or control vector were treated with CCCP (5 µM) for 2 h. YFP-Parkin (green), Tom20 (red), Flag-PTEN-L (cyan). Scale bar, 10 µm. **b** Percentage of cells with Parkin mitochondrial translocation was quantified by counting at least 300 cells. **c** Wild-type (WT) and *PTEN-L* KO YFP-Parkin-HeLa cells were treated with CCCP (4 µM) for 30 and 90 min. YFP-Parkin (green). Scale bar, 10 µm. **d** Percentage of cells with Parkin mitochondrial translocation from **c** (CCCP 90 min) was quantified by counting at least 300 cells. **e** *PTEN-L* KO YFP-Parkin-HeLa cells were transiently transfected with mCherry-PTEN-L or control vector and treated with CCCP (4 µM) for 90 min. YFP-Parkin (green), mCherry (red). Scale bar, 10 µm. Data in **b**, **d** are presented as mean ± SD from three independent experiments. **P < 0.01, ***P < 0.001 (two-way ANOVA test)

Following mitochondrial depolarization, recruitment of Parkin to damaged mitochondria is critical for mitophagy.^7, 42,43^ Here, we examined whether PTEN-L could affect Parkin mitochondrial localization. Overexpression of PTEN-L effectively blocked Parkin mitochondrial translocation induced by CCCP (Fig. 3a, b and Supplementary information, Movie S1). Similar results were also obtained in cells treated with O/A (Supplementary information, Figure S5a and b). In contrast, transient expression of PTEN had no effect on Parkin translocation (Supplementary information, Figure S5c). Next, we used the *PTEN-L* knockout (KO) HeLa cells established earlier and found that deletion of *PTEN-L* expedited Parkin translocation to mitochondria as evident changes occurred as early as 30 min after CCCP treatment (Fig. 3c, d and Supplementary information, Movie S2). Expectedly, ectopic expression of PTEN-L in *PTEN-L* KO HeLa cells prevented Parkin mitochondrial translocation induced by CCCP (Fig. 3e). We also made use of *PTEN*-KO mouse embryonic fibroblasts (MEFs)^44^ in which *PTEN-L* is also deficient (Supplementary information, Figure S5d, left panel) and found that transient overexpression of PTEN-L in *PTEN*-KO MEFs effectively blocked CCCP-induced Parkin mitochondrial translocation (Supplementary information, Figure S5d, right panel).

### PTEN-L impairs Parkin E3 ligase activity and reduces pSer65-Parkin

**Fig. 4:**
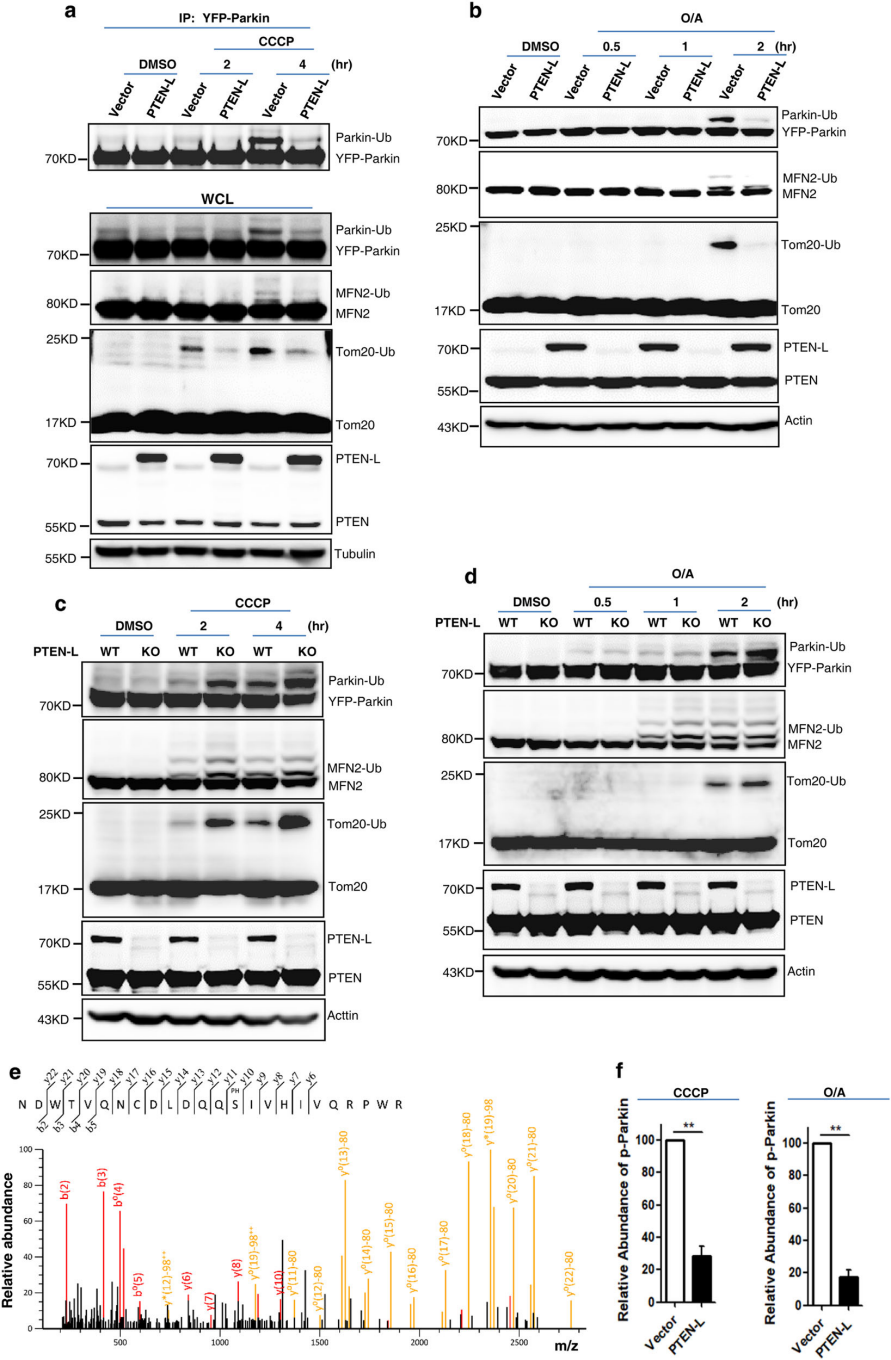
PTEN-L impairs Parkin E3 ligase activity and reduces pSer65-Parkin. **a** YFP-Parkin-HeLa cells with PTEN-L stable expression or control vector were treated with CCCP (5 µM) for indicated hours. YFP-Parkin was immunoprecipitated with anti-GFP antibody. Immunoprecipitants (IPs) and whole-cell lysates (WCLs) were analyzed for YFP-Parkin, mitofusin-2 (MFN2), Tom20, PTEN-L, PTEN and tubulin. **b** YFP-Parkin-HeLa cells with PTEN-L stable expression or control vector were treated with O/A (10 nM and 100 nM) for indicated hours and the WCLs were analyzed by immunoblotting as indicated. **c** Wild-type (WT) and *PTEN-L* KO YFP-Parkin-HeLa cells were treated with CCCP (5 µM) for indicated hours and the WCLs were analyzed by immunoblotting as indicated. **d** Wild-type (WT) and *PTEN-L* KO YFP-Parkin-HeLa cells were treated with O/A (10 nM and 100 nM) for indicated hours and the WCLs were analyzed by immunoblotting as indicated. **e** The MS/MS spectra of the Parkin peptide containing phospho-Ser65. YFP-Parkin-HeLa cells were treated with CCCP (10 µM) for 4 h. YFP-Parkin was pulled down with GFP beads and subjected to MS/MS analysis. **f** pSer65-Parkin was quantified using MS-based relative quantification analysis in YFP-Parkin-HeLa cells with or without PTEN-L stable expression after CCCP (10 µM) and O/A (25 nM and 250 nM) treatment. Data are presented as mean ± SD from 3 independent experiments. **P < 0.01 (Student’s *t*-test)

During induction of mitophagy, Parkin undergoes a series of changes, including binding to pSer65-Ub, recruitment to mitochondria, phosphorylation at its UBL domain (pSer65) by PINK1, activation of its E3 ligase activity and promotion of ubiquitination of OMM proteins, thus constituting the feedforward mechanism in mitophagy.^4^ In our study, we observed that the presence of PTEN-L significantly blocked the auto-ubiquitination of Parkin and the polyubiquitination of Parkin substrates MFN2 and Tom20 induced by CCCP (Fig. 4a) or O/A (Fig. 4b), indicating that overexpression of PTEN-L is able to impair Parkin E3 ligase activity. In contrast, Parkin E3 ligase activity was greatly enhanced in *PTEN-L* KO cells after treatment with CCCP (Fig. 4c) or with O/A (Fig. 4d). One possible mechanism for the inhibitory effects on the E3 ligase activity by PTEN-L is to reduce the phosphorylation of Parkin. To ascertain this, we performed two assays to examine the changes of p-Parkin. First, the Phos-Tag assay showed that PTEN-L reduced p-Parkin level in cells treated with CCCP (Supplementary information, Figure S6a). Second, we pulled down Parkin in HeLa cells with or without stable expression of PTEN-L after CCCP or O/A treatment (Supplementary information, Figure S6b) and the precipitated Parkin was subjected to tandem mass spectrometry (MS/MS). We successfully identified pSer65-Parkin in cells treated with CCCP with high confidence (Fig. 4e and Supplementary information, Figure S6c) or O/A (data not shown). More importantly, MS-based relative quantification analysis showed that PTEN-L markedly reduced the level of pSer65-Parkin in cells treated with CCCP or O/A (Fig. 4f). Finally, we found that PTEN-L overexpression or knockout had no evident effects on mitochondrial membrane potential or PINK1 protein level in cells treated with CCCP (Supplementary information, Figure S7a-e). Such findings thus rule out the possibility that PTEN-L has any direct effects on mitochondrial depolarization or directly affects PINK1 protein stability.

### PTEN-L keeps Parkin in closed conformation by enhancing the interaction of Parkin UBL and RING1 domains in a protein phosphatase activity-dependent manner

**Fig. 5:**
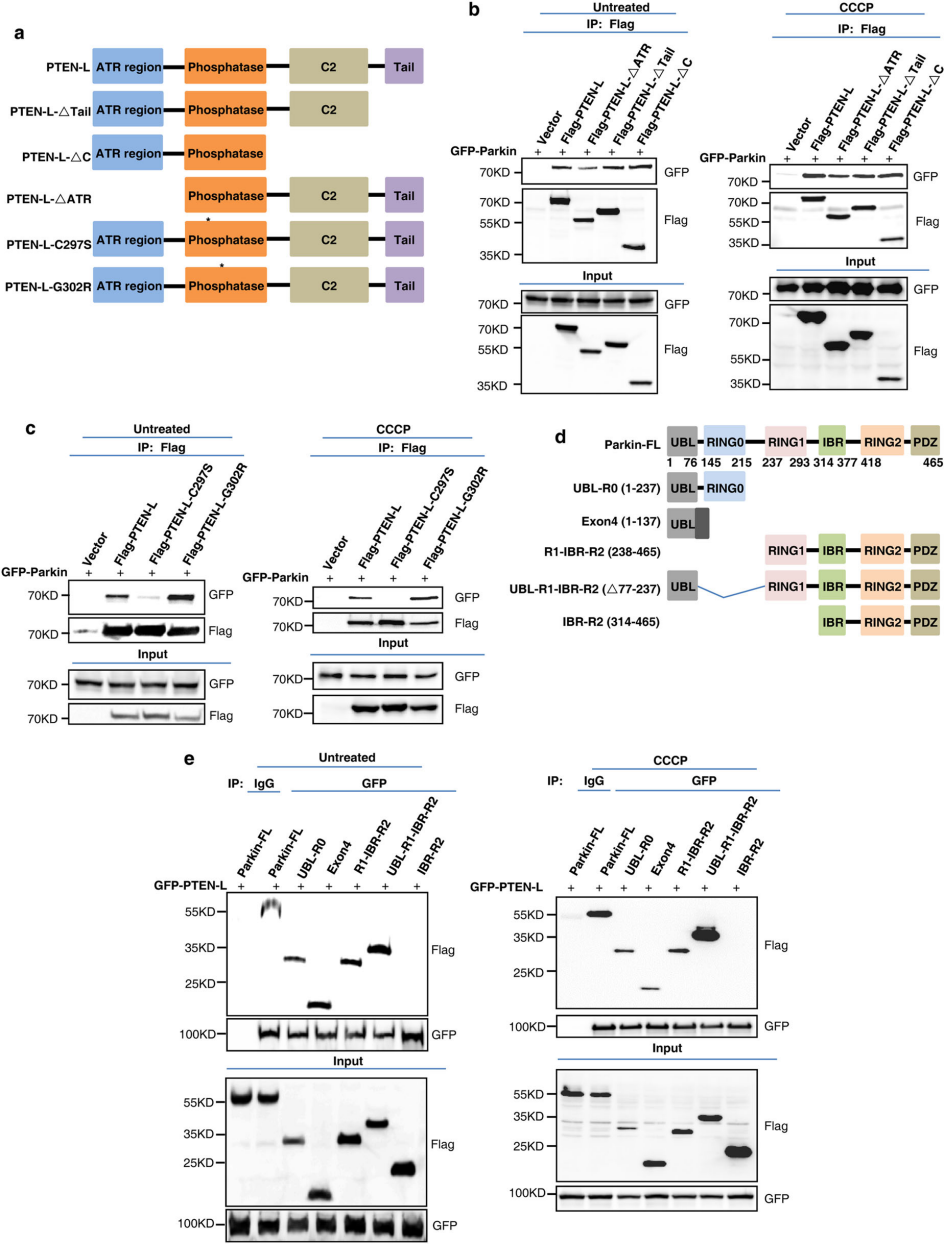

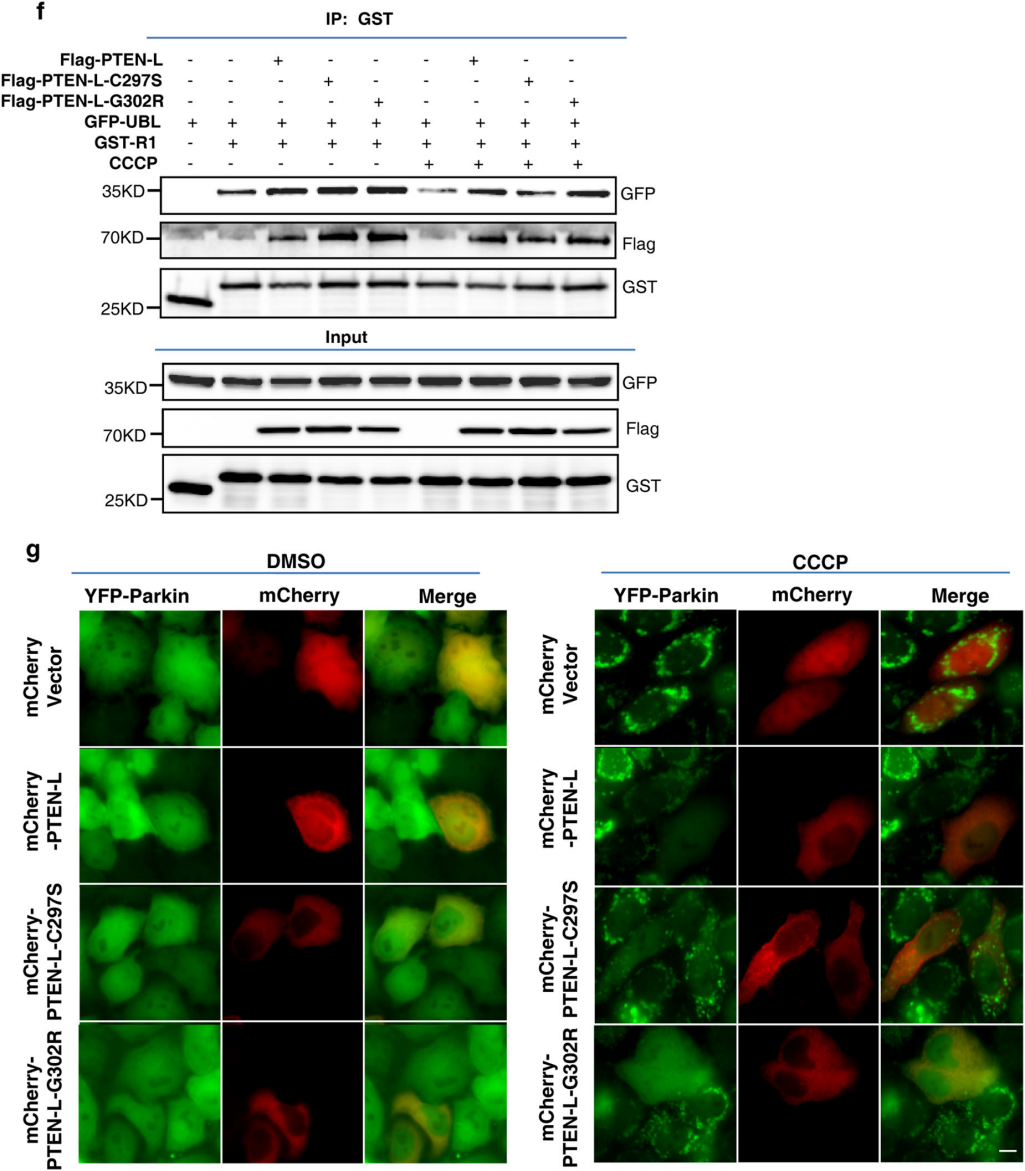
PTEN-L keeps Parkin in closed conformation by enhancing the interaction of Parkin UBL and RING1 domains in a protein phosphatase activity-dependent manner. **a** Construction of PTEN-L truncations. PTEN-L contains an ATR region, a phosphatase domain and a C-terminal region with a C2 domain and a C-Tail domain. PTEN-L-C297S is a dual lipid-protein phosphatase-defective mutant, while PTEN-L-G302R is a lipid phosphatase-defective mutant. **b**, **c** HEK293T cells transfected with GFP-Parkin and different constructs of Flag-tagged PTEN-L were treated without or with CCCP (5 µM) for 4 h. PTEN-L was immunoprecipitated with anti-Flag beads followed by immunoblotting for GFP and Flag. **d** Construction of Flag-tagged Parkin truncations, including Parkin-FL (full length) and truncated Parkin constructs: UBL-R0, Exon4, R1-IBR-R2, UBL-R1-IBR-R2 (deletion of R0 domain) and IBR-R2. **e** HEK293T cells transfected with GFP-PTEN-L and different constructs of Flag-tagged Parkin were treated without or with CCCP (5 µM) for 4 h. PTEN-L was then immunoprecipitated with anti-GFP beads followed by immunoblotting for Flag and GFP. **f** HEK293T cells were transfected with Flag-PTEN-L or the two Flag-PTEN-L mutants, together with Parkin truncation mutants GFP-UBL and GST-RING1 (R1) or GST empty vector (pEBG). Cells were then treated with or without CCCP (20 µM) for 4 h. RING1 was immunoprecipitated with anti-GST beads followed by immunoblotting for GFP, Flag and GST. **g** YFP-Parkin-HeLa cells transiently transfected with mCherry-PTEN-L or the two mCherry-PTEN-L mutants were treated with CCCP (5 µM) for 2 h. YFP-Parkin (green), mCherry (red). Scale bar, 10 µm

To further elucidate the molecular mechanisms underlying the regulatory function of PTEN-L in mitophagy, we examined the patterns of interaction between PTEN-L and Parkin. Using two different Parkin antibodies, we detected significant interaction between endogenous Parkin and endogenous PTEN-L in mouse brain tissue (Supplementary information, Figure S8a). Moreover, CCCP treatment appeared to promote Parkin and PTEN-L interaction (Supplementary information, Figure S8b), probably due to the increased PTEN-L mitochondrial translocation shown earlier (Supplementary information, Figure S1c, d, f and g). To understand the nature of such interactions, we generated a series of truncated forms of PTEN-L (Fig. 5a). One important finding was that deletion of either the ATR region, the Tail domain or the C2 domain of PTEN-L did not affect its binding to Parkin (Fig. 5b), indicating that the phosphatase domain of PTEN-L is required for this interaction. Moreover, we utilized two phosphatase-defective mutants of PTEN-L as reported previously.^33,34^ As expected, wild-type (WT) PTEN-L and the lipid phosphatase mutant (PTEN-L-G302R), but not the dual protein and lipid phosphatase mutant (PTEN-L-C297S), were able to reduce p-CREB, a known substrate for PTEN protein phosphatase activity^29^ (Supplementary information, Figure S8c). Interestingly, the PTEN-L-C297S mutant showed an impaired interaction with Parkin in cells with or without CCCP treatment (Fig. 5c), indicating the importance of the protein phosphatase activity of PTEN-L in mediating such interactions. Meanwhile, we also observed that the lipid phosphatase mutant PTEN-L-G302R had an increased interaction with Parkin, compared to the wild-type PTEN-L (Fig. 5c). The exact reason for this change remains to be further studied. To gain further insights into the interaction between PTEN-L and Parkin, we generated a set of Parkin truncation constructs (Fig. 5d) and found that the UBL and RING1 domains of Parkin are required for its interaction with PTEN-L (Fig. 5e and Supplementary information, Figure S8d and e). It is known that Parkin UBL domain binds to the RING1 domain to keep Parkin in its closed inactive status.^45–49^ We then investigated the effect of PTEN-L on the interaction of these two domains. Under basal condition (without CCCP treatment), wild-type PTEN-L and its two mutants had similar effects on the interaction of Parkin UBL and RING1 domains (Fig. 5f, left panel). Interestingly, wild-type PTEN-L as well as the lipid phosphatase mutant (PTEN-L-G302R) could stabilize the interaction between Parkin UBL and RING1 domains when mitochondria were depolarized by CCCP, whereas its dual phosphatase mutant (PTEN-L-C297S) failed to offer the same effects (Fig. 5f, right panel). Consistently, PTEN-L-C297S could not inhibit Parkin mitochondrial translocation after mitochondria damage induced by CCCP or O/A (Fig. 5g and Supplementary information, Figure S8f). Our data thus suggest that the protein phosphatase activity of PTEN-L is essential for inhibiting Parkin mitochondrial translocation. Since PINK1-mediated Parkin UBL phosphorylation (Ser65) is critical for Parkin activation via relieving the RING1 domain,^45–49^ it is very likely that PTEN-L could impair Parkin activation via reducing Parkin phosphorylation (pSer65) as shown earlier and stabilizing the interaction between Parkin UBL and RING1 domains, thus keeping Parkin in its closed inactive conformation.

### PTEN-L dephosphorylates ubiquitin

**Fig. 6:**
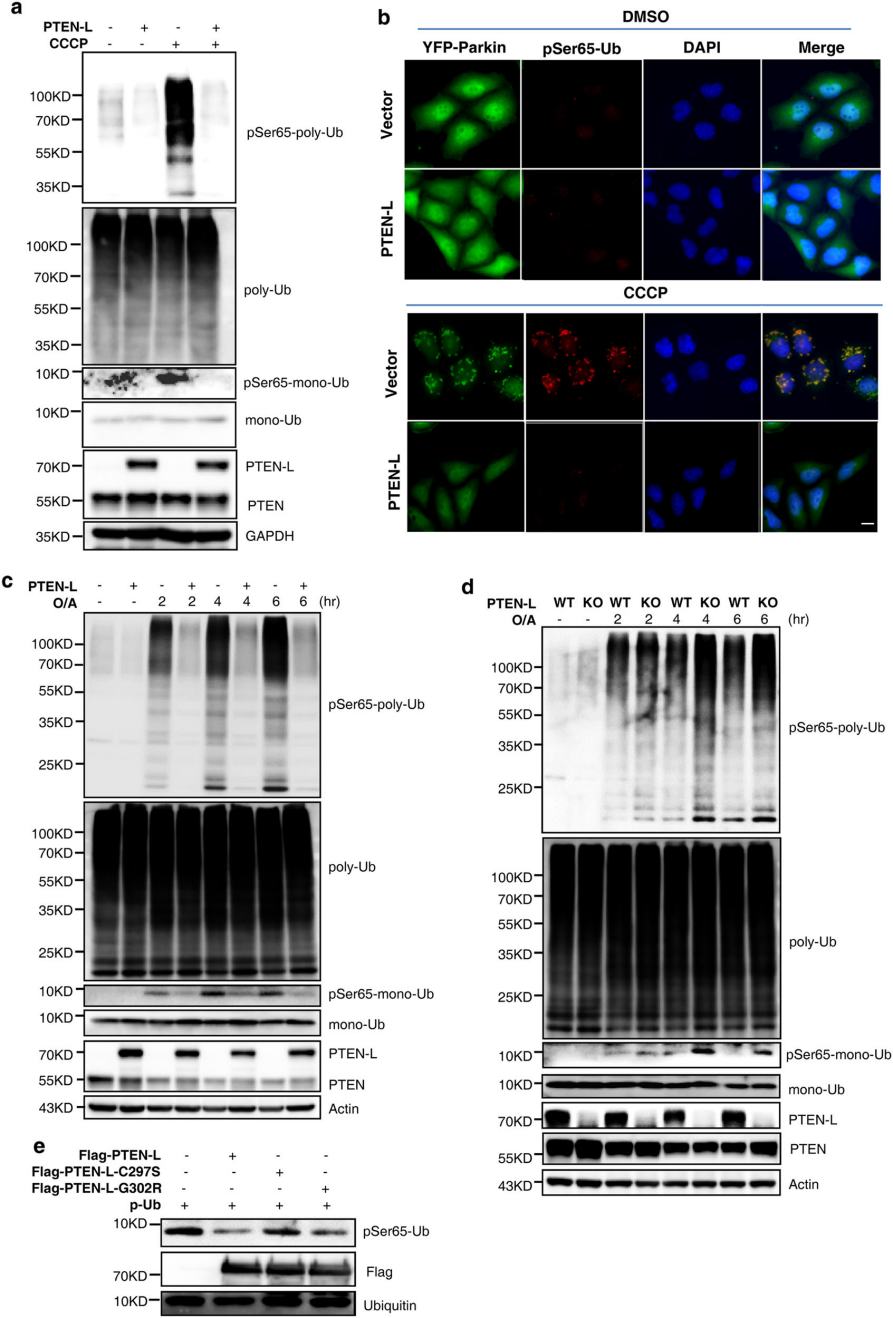
PTEN-L dephosphorylates ubiquitin. **a** YFP-Parkin-HeLa cells with PTEN-L stable expression or control vector were treated with CCCP (5 µM) for 3 h. Whole-cell lysates were analyzed by immunoblotting. **b** YFP-Parkin-HeLa cells with PTEN-L stable expression or control vector were treated with CCCP (5 µM) for 3 h. Immunofluorescence staining against pSer65-Ub was performed and observed by fluorescent microscopy. pSer65-Ub (red), YFP-Parkin (green), Nucleus (DAPI, blue). Scale bar, 10 µm. **c** YFP-Parkin-HeLa cells with PTEN-L stable expression or control vector were treated with O/A (25 nM and 250 nM) for indicated hours and immunoblotting was performed. **d** Wild-type (WT) and *PTEN-L* KO YFP-Parkin-HeLa cells were treated with O/A (25 nM and 250 nM) for indicated hours and immunoblotting was performed. **e** In vitro dephosphorylation assay. Purified pSer65-Ub was incubated with purified Flag-PTEN-L, Flag-PTEN-L-C297S or Flag-PTEN-L-G302R in phosphatase reaction buffer and ubiquitin phosphorylation level was evaluated by immunoblotting

PINK1-mediated phosphorylation of ubiquitin (pSer65-Ub) has been well established as a key event in PINK1–Parkin-mediated mitophagy.^10–12,41^ Since PTEN-L protein phosphatase activity is required for its inhibitory effect on Parkin mitochondrial translocation, we postulate that PTEN-L could serve as a direct phosphatase for pSer65-Ub. Indeed, pSer65-Ub was dramatically reduced in cells overexpressing PTEN-L after CCCP treatment as measured by immunoblotting (Fig. 6a) and immunohistochemistry (Fig. 6b). Similar results were obtained in cells with O/A treatment (Fig. 6c and Supplementary information, Figure S9a). In contrast, pSer65-Ub level was significantly increased in *PTEN-L* KO cells after O/A treatment (Fig. 6d). Accordingly, the in vitro phosphatase assays showed that both the purified PTEN-L and lipid phosphatase mutant (PTEN-L-G302R), but not the dual phosphatase mutant (PTEN-L-C297S), markedly reduced the pSer65-Ub level in vitro (Fig. 6e and Supplementary information, Figure S9b). Similar results were obtained when PTEN-L and its two mutants were transiently overexpressed in MEFs treated with CCCP (Supplementary information, Figure S9c). To further confirm the role of ubiquitin phosphorylation status in the effect of PTEN-L on Parkin translocation, we utilized two ubiquitin phospho-mutants, phosphorylation-deficient mutant (Ub-S65A) and phosphomimetic mutant (Ub-S65D). We found that overexpression of Ub-S65A alone was sufficient to block Parkin mitochondrial translocation in cells treated with O/A (Supplementary information, Figure S10a). Such findings were indeed consistent with the earlier reports.^10,11^ More importantly, Ub-S65D was able to override the inhibitory effect of PTEN-L on Parkin mitochondrial translocation (Supplementary information, Figure S10a), further supporting the notion that the inhibitory effect of PTEN-L on Parkin mitochondrial translocation is via ubiquitin dephosphorylation.

### PTEN-L disrupts the feedforward mechanism in mitophagy by targeting the pSer65-Ub chains

**Fig. 7:**
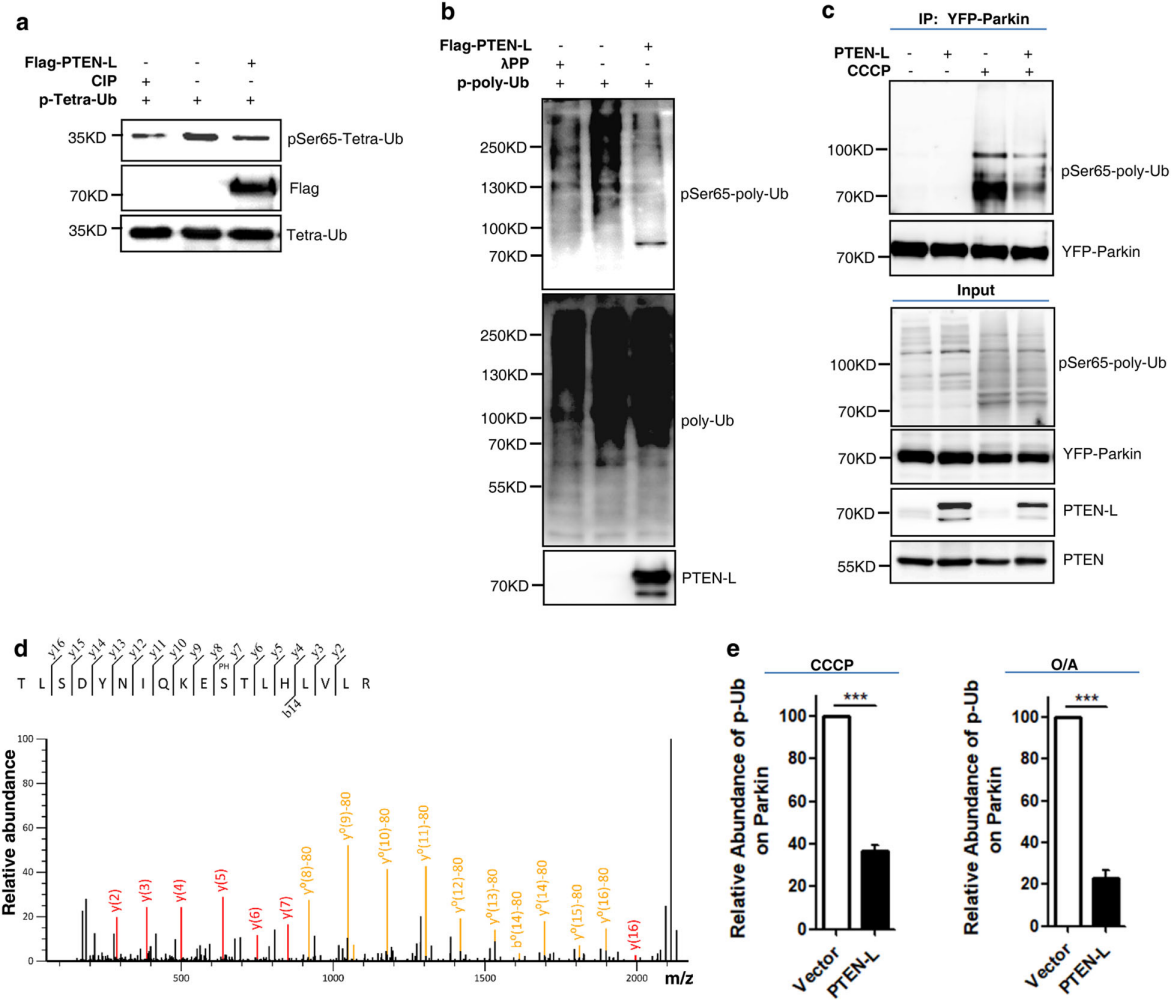
PTEN-L disrupts the feedforward mechanism in mitophagy by targeting the pSer65-Ub chains. **a** In vitro dephosphorylation assay using purified pSer65-tetra-Ub. Purified Flag-PTEN-L was incubated with pSer65-tetra-Ub in the phosphatase reaction buffer for 1 h at 30 °C. Calf intestinal phosphatase (CIP) was used as a positive control. **b** In vitro dephosphorylation assay using purified pSer65-poly-Ub chains, following the same procedure in **a**. λPP was used as a positive control. **c** YFP-Parkin-HeLa cells with PTEN-L stable expression or control vector were treated with CCCP (10 µM) for 4 h. YFP-Parkin was pulled down by GFP beads and subjected to immunoblotting. **d** The MS/MS spectra of the ubiquitin peptide containing phospho-Ser65. YFP-Parkin-HeLa cells were treated with CCCP (10 µM) for 4 h and YFP-Parkin was pulled down with GFP beads. **e** pSer65-Ub was quantified using MS-based relative quantification analysis in YFP-Parkin-HeLa cells with or without PTEN-L stable expression after CCCP (10 µM) and O/A (25 nM and 250 nM) treatment. Data are presented as mean ± SD from 3 independent experiments. ***P < 0.0001 (Student’s *t*-test)

Another important understanding in the molecular mechanisms of mitophagy is that the pSer65-Ub chains serve as a key receptor and activator for Parkin.^16–18, 50^ Here we further studied whether PTEN-L is able to target the pSer65-Ub chains. We first conducted the in vitro phosphatase assay and found that PTEN-L markedly reduces the level of pSer65-tetra-Ub (Fig. 7a) and pSer65-poly-Ub (Fig. 7b). Second, we observed the changes of phosphorylation status of poly-Ub chains binding to Parkin in the following two approaches. First, in cells treated with CCCP, PTEN-L markedly reduced the amount of pSer65-poly-Ub immunoprecipitated with Parkin (Fig. 7c). Second, the immunoprecipitants obtained via Parkin pull-down were subjected to MS/MS analysis and pSer65-poly-Ub was identified with very high confidence (Fig. 7d and Supplementary information, Figure 10b). Importantly, MS-based relative quantification analysis clearly showed that PTEN-L markedly reduced the pSer65-poly-Ub level in cells treated with CCCP or O/A (Fig. 7e). Thus, data from this part of our study clearly demonstrate that PTEN-L is able to directly dephosphorylate the pSer65-Ub chains, leading to the disruption of the feedforward loop between phosphorylated ubiquitin and Parkin to block mitophagy.

## Discussion

**Fig. 8:**
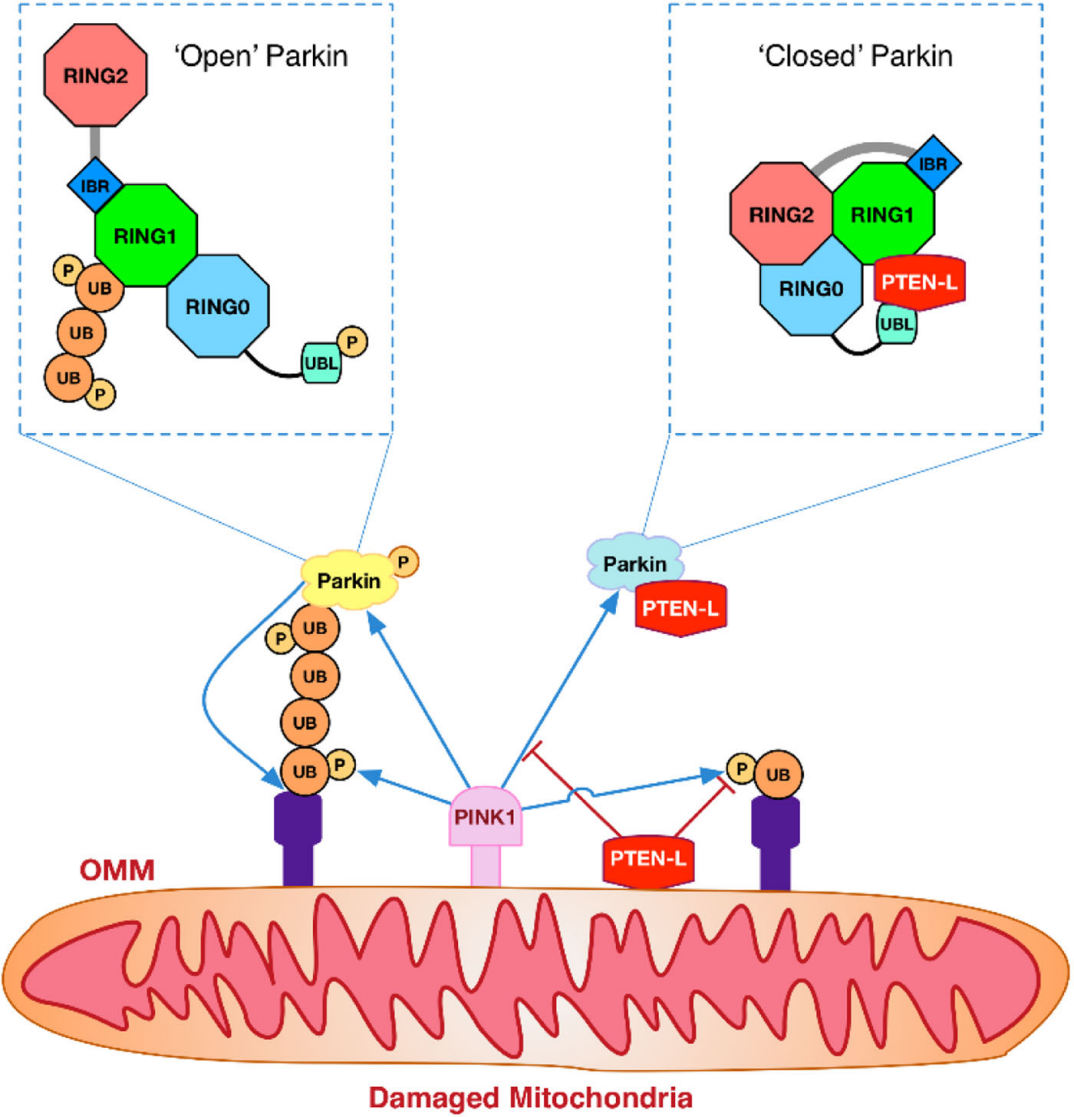
Illustration of the novel function of PTEN-L as a protein phosphatase in suppression of mitophagy. PTEN-L is able to counteract PINK1-mediated ubiquitin phosphorylation (pSer65-Ub), which then leads to prevention of Parkin recruitment to damaged mitochondria, reduction of pSer65-Parkin level and maintenance of Parkin in its closed inactive conformation to impair Parkin E3 ligase activity, all resulting in the disruption of the feedforward loop to inhibit mitophagy

The molecular mechanisms of PINK1–Parkin-mediated mitophagy have been extensively studied with relatively well-established models.^5, 6^ Briefly, the whole process of mitophagy induction consists of the following key steps. First, after mitochondrial damage or depolarization, PINK1 is stabilized on the OMM; PINK1 then mediates the phosphorylation of pre-existing ubiquitin, which drives Parkin mitochondrial translocation. Subsequently, PINK1 phosphorylates Parkin, changes its conformation and promotes its E3 ligase activity. Finally, PINK1-mediated ubiquitin and Parkin phosphorylation and Parkin-mediated ubiquitination of OMM proteins including Parkin itself constitute the feedforward mechanism, leading to the robust onset of mitophagy. From this process, it is obvious that PINK1-mediated phosphorylation and Parkin-mediated ubiquitination are the two key molecular events positively regulating mitophagy. In this study, we identified PTEN-L as a novel protein phosphatase that specifically targets PINK1-mediated phosphorylation of ubiquitin to suppress several of the above-mentioned key steps in mitophagy, as summarized in Fig. 8. PTEN-L serves as the phosphatase to dephosphorylate pSer65-Ub mediated by PINK1, which is the key step for blocking the subsequent events including Parkin translocation and phosphorylation, Parkin conformational changes and Parkin E3 ligase activation, eventually disrupts the feedforward loop and inhibits mitophagy. Thus, our data fill one key missing piece in molecular puzzle of mitophagy by identifying the long-awaited protein phosphatase balancing the effect of PINK1 in regulation of mitophagy.

In establishing the novel function of PTEN-L in regulation of mitophagy, we first examined the subcellular localization of PTEN-L via an array of experiments. We confirmed that a significant amount of PTEN-L is present in the mitochondria based on the data from cultured cells (Fig. 1b, c) and various mouse tissues (Supplementary information, Figure S1a), consistent with an earlier report.^33^ Importantly, we provided convincing evidence demonstrating that PTEN-L localizes at OMM based on results from the topology assay (Fig. 1d and Supplementary information, Figure S1e) and immune-gold EM (Fig. 1e), but not in the matrix as reported previously.^33^ It is believed that localization of PTEN-L at OMM provides the proximity for PTEN-L to target phosphorylated ubiquitin and Parkin that are accumulated at OMM upon mitochondrial damage. On the other hand, our data show that there is a significant amount of PTEN-L still in the cytosol. At this stage we could not rule out the possibility that the cytosolic PTEN-L may also target ubiquitin and/or Parkin for dephosphorylation, which leads to the suppression of mitophagy. Nevertheless, we believe that it is the mitochondria-localized PTEN-L that is playing a major role in dephosphorylating ubiquitin to inhibit mitophagy, based on the following two well-known facts. First, it is well known that upon mitochondrial damage, PINK1 is stabilized at the OMM and phosphorylates both ubiquitin and Parkin that are recruited to mitochondria.^8–12^ Second, previous studies have clearly demonstrated that phospho-ubiquitin is mostly accumulated at the OMM.^4,11,19,41,51^ The function of cytosolic PTEN-L needs to be further explored.

After establishing the negative regulatory effects of PTEN-L in mitophagy, we then attempted to reveal the underlying molecular mechanisms by which PTEN-L inhibits mitophagy. In doing so, we first focused the effects of PTEN-L on Parkin and examined the changes of the following three key processes: Parkin mitochondrial translocation, its E3 ligase activity and phosphorylation status. Overexpression of PTEN-L inhibits, while deletion of *PTEN-L* expedites, Parkin mitochondrial translocation after CCCP or O/A treatment (Fig. 3, Supplementary information, Figure S5, Movies S1 and 2), whereas overexpression of PTEN fails to provide similar effects. More importantly, overexpression of PTEN-L significantly impairs Parkin E3 ligase activity as evidenced by reduced Parkin auto-ubiquitination or polyubiquitination of its substrates (Fig. 4a, b). Consistently, deletion of *PTEN-L* promotes Parkin E3 ligase activity (Fig. 4c, d). It is well known that there are two major factors regulating Parkin E3 ligase activity, binding to pSer65-Ub and phosphorylation by PINK1. These two events result in the conformational changes of Parkin from its closed inactive structure to opened active status.^16–18,45–47,50^ Another important observation of our study is the marked reduction of pSer65-Parkin level upon overexpression of PTEN-L (Fig. 4e, f and Supplementary information, Figure S6). There are two possibilities for the reduced pSer65-Parkin level in the presence of PTEN-L. First, PTEN-L may serve as the direct protein phosphatase against pSer65-Parkin mediated by PINK1, similar to the effect on pSer65-Ub. Second, a more likely possibility is that the reduced pSer65-Parkin level in the presence of PTEN-L is a secondary event following reduced pSer65-Ub level at OMM, which impairs Parkin mitochondrial translocation and eventually less phosphorylation by PINK1. Future work is needed to confirm whether PTEN-L could serve as a direct phosphatase for pSer65-Parkin.

In the current model of PINK1–Parkin-mediated mitophagy, phosphorylation of ubiquitin by PINK1 is considered as a key step in triggering mitophagy.^4,5^ For instance, PINK1 is able to phosphorylate pre-existing ubiquitin at mitochondria without the involvement of Parkin.^41^ Moreover, pSer65-Ub serves as a potent receptor for recruiting Parkin to mitochondria,^16,50^ and binding of pSer65-Ub to Parkin is able to change Parkin conformations and activates its E3 ligase activity.^46,47^ In our study we conducted a series of experiments to examine the effect of PTEN-L on pSer65-Ub and we concluded that PTEN-L is a direct protein phosphatase against PINK1-mediated phosphorylation of ubiquitin, based on evidence from multiple in vivo and in vitro qualitative and quantitative assays. More importantly, we also provide strong and consistent evidence demonstrating the effects of PTEN-L on pSer65-Ub chains, a key element in the feedforward mechanism in mitophagy, as discussed earlier. For instance, overexpression of PTEN-L reduces pSer65-Ub chains (Fig. 6a, c) and knockout of *PTEN-L* increases pSer65-Ub chains (Fig. 6d) in vivo; PTEN-L dephosphorylates pSer65-mono-Ub, pSer65-tetra-Ub and pSer65-poly-Ub chains in vitro (Figs. 6e and 7a, b); and importantly, PTEN-L reduces the binding of pSer65-poly-Ub chains to Parkin (Fig. 7c–e). Taken together, it is clear that PTEN-L serves as a direct protein phosphatase against PINK1-mediated ubiquitin phosphorylation, leading to the blockage of a series of sequential steps, including the feedforward mechanism to suppress mitophagy.

In summary, our study provides a key missing piece in the molecular puzzle controlling mitophagy by identifying the novel function of PTEN-L as a protein phosphatase against PINK1-mediated ubiquitin phosphorylation. Thus, the balance of PTEN-L and PINK1 activity may be critical for maintaining mitochondrial homeostasis. Defective mitophagy is implicated in human diseases such as neurodegenerative disorders, in particular Parkinson’s disease. Therefore, our findings may provide some important clues for development of novel interventional strategies by targeting PTEN-L for reactivation of mitophagy.

## Materials and methods

### Reagents and antibodies

CCCP, oligomycin, antimycin A, valinomycin, G418, puromycin, calf intestinal alkaline phosphatase and FLAG^®^ peptide were purchased from Sigma. Lipofectamine 3000, 4′,6-diamidino-2-phenylindole (DAPI) and protein A/G Plus agarose were from Thermo Fisher Scientific. Lambda Protein Phosphatase (λPP) was from New England Biolabs. Phos-tag™ acrylamide AAL-107 was from Wako. HyperSignal ECL substrate was purchased from 4A Biotech Co., Ltd. Amersham ECL prime detection reagent was from GE Healthcare.

Antibodies against the following proteins were from Cell Signaling Technology: PTEN (138G6, 9559), GAPDH (2118), Parkin (4211), AKT (9272), pAKT473 (9271), GFP (2956), PINK1 (6946), GST (2624), CREB (9197), pCREB133 (9198), mitofusin-2 (MFN2, 11925), mitofusin-1 (MFN1, 14739), rabbit IgG (3900) and mouse IgG (5415). The following antibodies were from Sigma: anti-Flag (F1804), anti-actin (A5441) and anti-tubulin (T6199). Antibodies to Tom20 (FL-145), HA (7392) and ubiquitin (P4D1, 8017) were from Santa Cruz. Anti-Tim23 (611223) antibody was from BD Biosciences. Phospho-ubiquitin (ABS1513-I) and anti-PTEN-α (anti-PTEN-L, MABS1680) antibodies were from Merck Millipore. Anti-DNA (61014) was from Progen Biotechnik. Antibodies to COX II (ab110258) and Calreticulin (ab92516) were from Abcam.

The following secondary antibodies were from Thermo Fisher Scientific: Alexa405 goat anti-mouse (A-31553), Alexa488 anti-mouse (A-32723), Alexa488 anti-rabbit (A-11034), Alexa592 goat anti-mouse (A-11032), Alexa592 goat anti-rabbit (R37117) and Alexa633 goat anti-rabbit (A-21072). Peroxidase-conjugated affinity pure goat anti-mouse IgG, light chain specific (115-035-174) and peroxidase-conjugated IgG fraction monoclonal mouse anti-rabbit, light chain specific (211-032-171) were purchased from Jackson ImmunoResearch.

### Plasmid constructs

PTEN-L and PTEN complementary DNAs (cDNAs) were generated by reverse transcription PCR from RNAs of HeLa cells and inserted into pCMV-Tag2B vector with an N-terminal Flag tag or pcDNA3 vector with an N-terminal HA tag. Subcloning was performed to generate a different set of constructs of Flag-PTEN-L, GFP-PTEN-L (pEGFP-C1 vector) and mCherry-PTEN-L (pmCherry-C1 vector). All the Flag-Parkin truncations were generated in pcDNA3.1 vector and GFP-Parkin was generated by inserting Parkin into pEGFP-C1 vector. mCherry-Parkin plasmids were kind gifts from Dr. Richard Youle (National Institute of Neurological Disorders and Stroke). GFP-Parkin UBL domain and GST-Parkin-RING1 domain plasmids were kind gifts from Dr. Jongkyeong Chung (Seoul National University).

Site-directed mutagenesis was performed by using the QuickChange Site-Directed Mutagenesis Kit following the manufacturer’s instructions. All plasmid constructs were confirmed by restriction digestion or DNA sequencing. Expression of all constructs was confirmed by immunoblotting.

### Cell lines, cell culture and transfections

Cells were maintained in Dulbecco’s modified Eagle’s medium (Sigma, D7777) with 10% fetal bovine serum (Hyclone, SH30071.03) and 1% penicillin–streptomycin (Pan-Biotech, P06-07100) in a 5% CO_2_ incubator at 37 °C. Transfections with plasmid DNA were performed with Lipofecamine 3000 according to the manufacturer’s instructions when cells were grown to 70% confluency.

HeLa cells stably expressing YFP-Parkin were kind gifts from Dr. Richard Youle. *PTEN*-knockout MEF was a kind gift from Dr. Tak W. Mak (University of Toronto) and Dr. Thilo Hagen (National University of Singapore).

To generate HeLa cells stably expressing YFP-Parkin and Flag-PTEN-L (YFP-Parkin-HeLa cells with PTEN-L stable expression), YFP-Parkin-expressing HeLa cells were transiently transfected with pCMV-Tag2B harboring full-length PTEN-L. After 24 h of transfection, selection reagent G418 (700 µg/ml) was added to the cells in fresh medium. After 10 days, cells were digested with Trypsin (Thermo Fisher Scientific) and replated at a low density with selection reagent (G418, 700 µg/ml). After an additional 7 days of incubation, single clones were picked up and replated in a new dish with fresh medium. To maintain the resistant phenotype of established transfected cells, stable cell lines were maintained in medium with 200 µg/ml G418.

All other cell lines used in this study were purchased from American Type Culture Collection. All cells were tested for mycoplasma contamination bimonthly using the MycoAlert PLUS kits from Lonza.

### Mitochondria fractionation and topology analysis

Mitochondria were isolated by differential centrifugation as described previously.^38^ Topology analyses of PTEN-L were performed by proteinase K protection assay as described previously^38^ with some modifications. Immediately after isolation, the mitochondrial pellet was re-suspended in MIM buffer (280 mM sucrose with 10 mM HEPES, pH 7.2). Mitochondria (40 µg per condition) were placed in MIM buffer containing varying concentrations of digitonin (0–0.2%) and constant concentration of proteinase K (100 µg/ml) for 15 min on ice. Samples without proteinase K or with 1% Triton X-100 served as controls. Proteinase K was inactivated by addition of 10 mM phenylmethylsulfonyl fluoride and samples were subjected to western blotting.

All the animal work procedures were approved by the Institutional Animal Care and Use Committee of National University of Singapore (Protocol# R13–05884).

### *PTEN-L*-KO cell generation by CRISPR/Cas9

To specifically knock out *PTEN-L* but not affect *PTEN* expression, the sgRNAs were designed to target the DNA sequence between CTG and ATG in the exon 1 of *PTEN-L* based on the Zhang laboratory website (http://crispr.mit.edu/). Complementary oligonucleotides encoding gRNAs were annealed and cloned into *Bsm*BI (Fermentas) sites in lentiCRISPRv2 (Addgene). Lentiviral particles were generated by transfecting HEK293T cells with lentiCRISPRv2-gRNA construct, psPAX2 and pMD2.G (Addgene) at a ratio of 4:3:1. Viral supernatants were collected 48–72 h following transfection and concentrated using the centrifugal filter (Merck Millipore) according to the manufacturer’s protocol. YFP-Parkin-expressing HeLa cells were transduced with lentivirus. After 24 h of infection, cells were detached with TrypLE (Thermo Fisher Scientific) and replated at low density. After 3 h of plating, cells were treated with selection agent (1 µg/ml puromycin, Sigma). After 7 days, puromycin-resistant cells were harvested to detect *PTEN-L* knockout by T7E1 assays and immunoblotting. Then, single cell cloning was performed accordingly and maintained in medium with 1 µg/ml puromycin.

sgRNA sequence: 5′-CGGCGGCACATCCAGGGACC-3′.

PCR primers:

Forward: 5′-CTTCCTCGGCTTCTCCTGAAA-3′

Reverse: 5′-CTAAGTCGAATCCATCCTCTT-3′.

### Transmission electron microscopy and immunogold EM

YFP-Parkin-HeLa cells with PTEN-L stable expression or with vector control were fixed for 4 h at 4 °C with freshly prepared fixative mixture (2% paraformaldehyde + 3% glutaraldehyde) in 0.1 M cacodylate buffer, pH 7.4, and then rinsed 3 times with the same buffer. Samples were post-fixed with 2% OsO_4_ (pH 7.4) for 1 h at room temperature followed by block-staining and washing twice with 0.1 M phosphate buffer (PB). Samples were then dehydrated through an ascending ethanol series (25%, 50%, 75%, 95%, 100%) followed by 100% acetone for 10 min twice, and embedded in fresh resin and polymerized at 60 °C for 24 h. Ultrathin sections were prepared and stained with uranyl acetate and lead citrate, rinsed with distilled water and observed on an electron microscope (JEOL, JEM-1010).

To perform immunogold electron microscopy (EM) assay, HeLa-YFP-Parkin cells stably expressing PTEN-L were treated without or with CCCP (5 µM) for 4 h. Cells were fixed in 4% paraformaldehyde and 0.2% glutaraldehyde in 0.1 M PB at room temperature for 1 h. Any remaining aldehyde was quenched with 0.05 M glycine in PB and samples were blocked in 1% bovine serum albumin (BSA) + 0.05% TX-100 + 0.1% CWFG in 0.1 M PB for 30 min. Immunoblotting with anti-FLAG (1:100; Sigma, F7425) was performed overnight at 4 °C with gentle agitation, followed by incubation with goat anti-rabbit IgG coupled to 5 nm gold particles (1:20; Sigma, C7277). Samples were rinsed with PBS–BSA (3× 1 min), then PBS (3× 1 min) and finally with deionized water (2× 1 min). The Silver Enhancer reagent was prepared and applied following the manufacturer’s instructions (Abcam, 170732). After silver enhancement, samples were rinsed twice in deionized water for 5 min. Samples were post-fixed in OsO_4_ and dehydrated in a series of ethanol washes (25%–100%), embedded in Araldite and observed on an electron microscope (JEOL, JEM-1010).

### Immunofluorescence and time-lapse microscopy

Cells were grown overnight on coverslips before treatment as indicated. Cells were washed with PBS, fixed with 4% paraformaldehyde in PBS for 15 min at room temperature, permeabilized with 0.25% Triton X-100 for 15 min and blocked in 10% BSA in PBS for 30 min at 37 °C before being stained with the indicated antibodies. Coverslips were mounted with ProLong® Diamond Antifade Mountant (Thermo Fisher Scientific), and observed on a confocal microscope (Olympus FV3000 Confocal Laser Scanning Microscope) or a fluorescence microscope (Leica, DMi8). Percentage of cells with YFP-Parkin mitochondrial translocation was quantified from a minimum of 300 cells.

To measure mitophagy by mtDNA, immunostaining was performed as described previously^41^ with some modifications. Ten image slices were collected on an Olympus FV3000 Confocal Laser Scanning Microscope through the Z plane encompassing the top and bottom of the cells. Image analysis was performed on all images collected (> 300 cells from three independent experiments) using Imaris 9.1 software. The mtDNA stain volume in vector control untreated cells was normalized to 100% and the amount of mtDNA stain remaining after CCCP treatment was subsequently determined.

To analyze the colocalization of PTEN-L with Tom20, PTEN with Tom20 and PTEN-L with Calreticulin, at least 5 different fields were imaged through the Z plane using an Olympus FV3000 Confocal Laser Scanning Microscope. The confocal images were acquired by 60×/1.3 oil lens for high resolution with pixel size of 87 nm in lateral direction and 120 nm in axial direction, respectively. Huygens deconvolution was applied to all the images for better signal-to-noise ratio. Colocalization channels (in white color) were built by using Imaris (9.01 version) software Colocalization module by applying automatic selection of the thresholds to get the user bias out of the equation, and then Pearson’s coefficient in region of interest volume was assessed.

YFP-Parkin-expressing HeLa cells were plated in glass chamber slides (ibidi) and left to adhere overnight followed by transfection with plasmid encoding mCherry-PTEN-L. After 24 h, cells were treated with CCCP (5 µM). Imaging was started immediately after adding CCCP. XY-stacks were acquired every minute for 2 h using a fluorescence microscope (Leica, DMi8) and exported as uncompressed AVI sequences at 13 frames per second. Imaging of Parkin translocation in YFP-Parkin-HeLa cells without (WT) or with *PTEN-L* knockout (KO) was performed as above.

### Immunoblotting and immunoprecipitation assays

Cells were washed in ice-cold PBS and lysed in lysis buffer (62.5 mM Tris, pH 6.8, 25% glycerol, 2% SDS, protease and phosphatase inhibitors, 1 mM dithiothreitol (DTT)). Cell lysates were subjected to sodium dodecyl sulfate–polyacrylamide gel electrophoresis (SDS-PAGE) and immunoblot analysis was performed accordingly. For quantification of the mitochondrial markers, grayscale values of corresponding bands were measured with ImageJ software, and the fold change was calculated by comparing the treated groups with the respective vector control dimethyl sulfoxide groups.

To analyze Parkin E3 ligase activity, lysates were prepared in sample buffer (20 mM Tris, pH 8.0, 150 mM NaCl, 0.5% NP-40, 1 mM EDTA, pH 8.0, and 10 mM NEM).

For immunoprecipitation assays, HEK293T cells were transfected with the indicated plasmids. After 24 h, cells were lysed with lysis buffer containing 150 mM NaCl, 1 mM EDTA, 0.5% NP-40, 50 mM Tris-HCl, pH 7.5, 1 mM NEM and protease and phosphatase inhibitors. Lysates were precleared with protein A/G agarose for 1 h at 4 °C. Precleared lysates were immunoprecipitated with the indicated antibodies overnight at 4 °C, and then incubated with protein A/G agarose for additional 2 h at 4 °C. The beads were washed four times with lysis buffer. The bound proteins were eluted by boiling and subjected to SDS-PAGE and immunoblotting.

### Phos-tag gels

To analyze Parkin phosphorylation, lysates were prepared in sample buffer lacking EDTA and run on 7.5% Tris-glycine gels containing 50 µM phos-tag (Wako) and 100 µM MnCl_2_ following the manufacturer’s instructions. SDS-PAGE gels were run concurrently as a negative control.

### In vitro dephosphorylation assay

HEK293T cells were transiently transfected with control vector or vectors encoding Flag-PTEN-L, Flag-PTEN-L-C297S and Flag-PTEN-L-G302R. After 24 h, cells were lysed with immunopreciptation buffer and subjected to Flag immunoprecipitation with anti-Flag M2 affinity gel (Sigma) overnight at 4 °C. Samples were washed 4 times with lysis buffer, and the Flag-PTEN-L, PTEN-L mutants and control protein were eluted with Flag peptide (400 µg/ml, Sigma) for 2 h at 4 °C. Purified phospho-Ser65-ubiquitin (Ubiquigent Ltd) or purified phospho-Ser65-tetra-ubiquitin chains (R&D Systems) were added to ubiquitin antibody (P4D1, Santa Cruz) and incubated overnight at 4 °C followed by incubation with protein A/G agarose for 2 h at 4 °C. Samples were washed twice with phosphatase reaction buffer containing 20 mM HEPES (pH 7.2), 1 mM DTT, 1 mM MgCl_2_, 0.1 mg/ml BSA and 1 mM EDTA with protease inhibitors. For dephosphorylation of p-poly-Ub chain, chelator-free reaction buffer containing 50 mM Tris (pH 7.5), 100 mM NaCl, 10 mM MgCl_2_, 1% glycerol, 10 µM ATP, and 10 mM DTT was used, followed by incubation at 30 °C for 1 h. The same amount of PTEN-L, PTEN-L mutants and control protein as well as calf intestinal alkaline phosphatase or λPP were added to the phosphatase reaction buffer containing phospho-ubiquitin, followed by incubation at 30 °C for 1 h. Beads were washed twice with phosphatase reaction buffer, and the bound proteins were eluted by boiling and subjected to SDS-PAGE and immunoblotting.

### Data availability

All data that support the findings of this study are included in the manuscript or are available from the authors upon reasonable request.

## Electronic supplementary material

Supplementary information, Figure S1

Supplementary information, Figure S2

Supplementary information, Figure S3

Supplementary information, Figure S4

Supplementary information, Figure S5

Supplementary information, Figure S6

Supplementary information, Figure S7

Supplementary information, Figure S8

Supplementary information, Figure S9

Supplementary information, Figure S10

Supplementary information, Movie S1

Supplementary information, Movie S2

Supplementary movie legend
