# Supplementary material for: PTEN-L is a novel protein phosphatase for ubiquitin dephosphorylation to inhibit PINK1–Parkin-mediated mitophagy
Source: Cell Res. 2018 Jun 22;28(8):787–802. doi: 10.1038/s41422-018-0056-0 (PMC6082900; doi:10.1038/s41422-018-0056-0)
Supplement: Supplementary file 1 — Supplementary information, Figure S1 [file 41422_2018_56_MOESM1_ESM.pdf]

Supplementary information, Figure S1

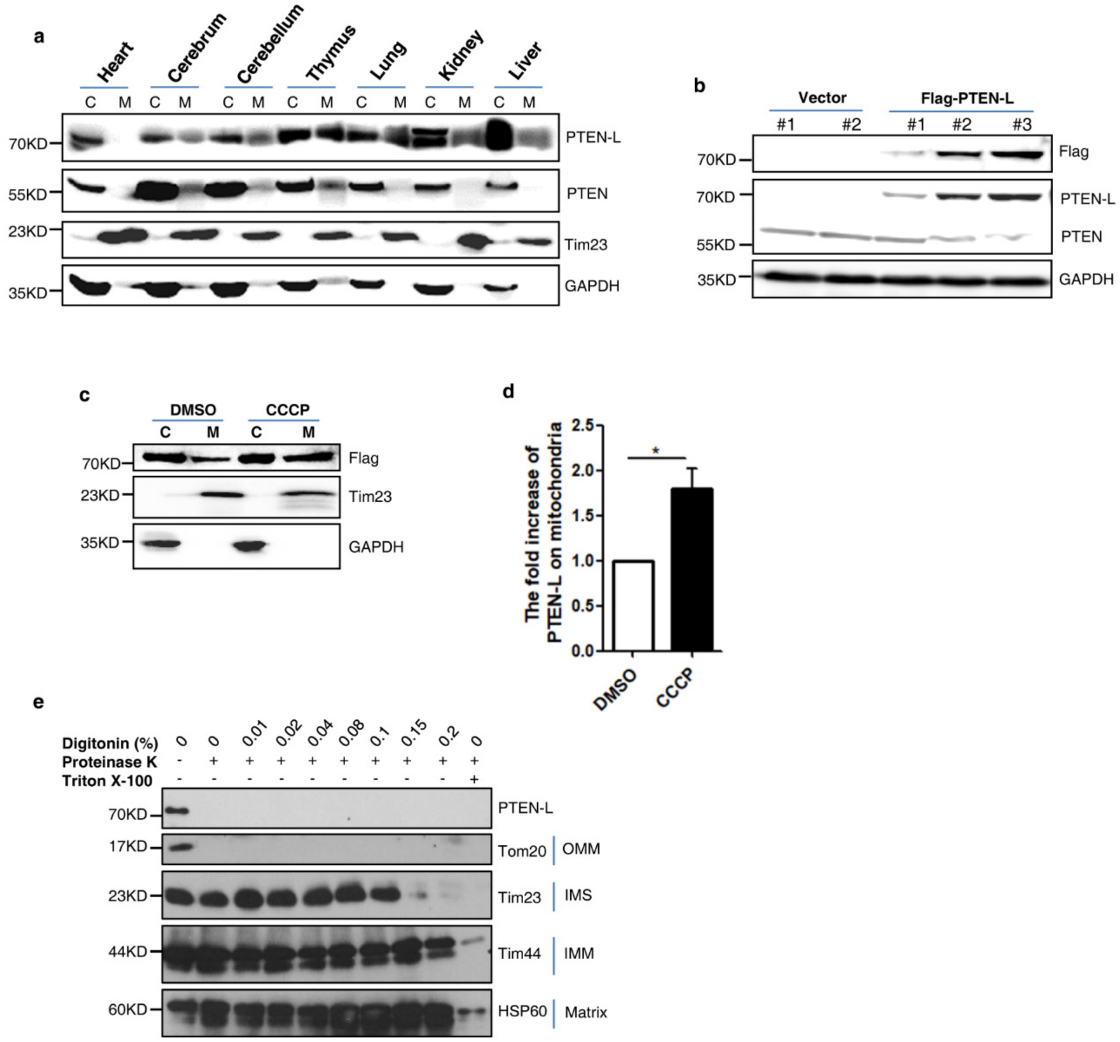

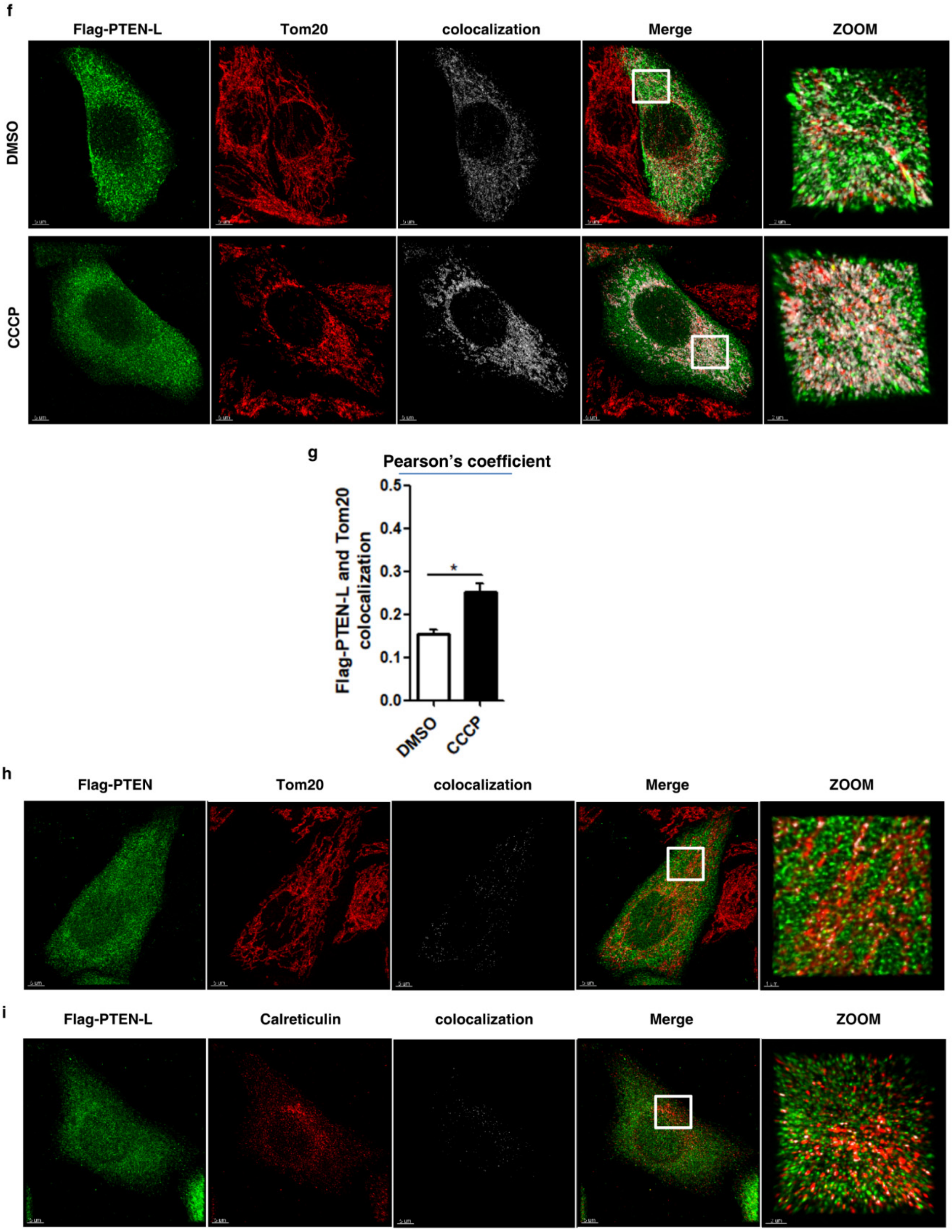
f
Flag-PTEN-L
Tom20
colocalization
Merge
ZOOM
DMSO
CCCP
g
Pearson's coefficient
Flag-PTEN-L and Tom20 colocalization
0.5
0.4
0.3
0.2
0.1
0.0
*
DMSO
CCCP
h
Flag-PTEN
Tom20
colocalization
Merge
ZOOM
i
Flag-PTEN-L
Calreticulin
colocalization
Merge
ZOOM

Figure S1 PTEN-L resides at the outer mitochondrial membrane. a Mitochondria fraction was isolated from various tissues in mouse and analysed with immunoblotting as indicated. Tim23 and GAPDH were used as mitochondrial and cytosolic markers, respectively. C, cytosol; M, mitochondria. **b** Establishment of YFP-Parkin-HeLa cells with PTEN-L stable expression. The expression of PTEN-L in three stable cell lines (#1, #2, #3) was detected by immunoblotting with Flag, PTEN (138G6) and GAPDH antibodies. Clone #2 was used for subsequent studies. **c** YFP-Parkin-HeLa cells with PTEN-L stable expression were treated with DMSO or CCCP (5 μM) for 4 h. Cell fractionation was conducted to isolate mitochondria and immunoblotting was performed. Tim23 and GAPDH were used as mitochondrial and cytosolic markers, respectively. C, cytosol; M, mitochondria. **d** Mitochondrial PTEN-L was quantified from **c** and data is presented as mean ± SD from 3 independent experiments. *$P < 0.05$ (Student's *t*-test). **e** Topology assay with isolated mitochondria from YFP-Parkin-HeLa cells treated with different doses of proteinase K and digitonin. **f** Co-localization of Flag-PTEN-L with Tom20 without or with CCCP (5 μM, 4 h treatment) was examined by confocal fluorescence microscopy. Flag-PTEN-L (Green); Tom20 (Red); colocalization channel (White). Scale bars, 5 μm and 1 μm (ZOOM). **g** Pearson's coefficient in ROI volume from **f**. *$P < 0.05$ (Student's *t*-test). **h** Co-localization of Flag-PTEN with Tom20. Flag-PTEN (Green); Tom20 (Red); colocalization channel (White). Scale bars, 5 μm and 1 μm (ZOOM). **i** Co-localization of Flag-PTEN-L with Calreticulin. Flag-PTEN-L (Green); Calreticulin (Red); colocalization channel (White). Scale bars, 5 μm and 1 μm (ZOOM).
