# Supplementary material for: PTEN-L is a novel protein phosphatase for ubiquitin dephosphorylation to inhibit PINK1–Parkin-mediated mitophagy
Source: Cell Res. 2018 Jun 22;28(8):787–802. doi: 10.1038/s41422-018-0056-0 (PMC6082900; doi:10.1038/s41422-018-0056-0)
Supplement: Supplementary file 2 — Supplementary information, Figure S2 [file 41422_2018_56_MOESM2_ESM.pdf]

# Supplementary information, Figure S2

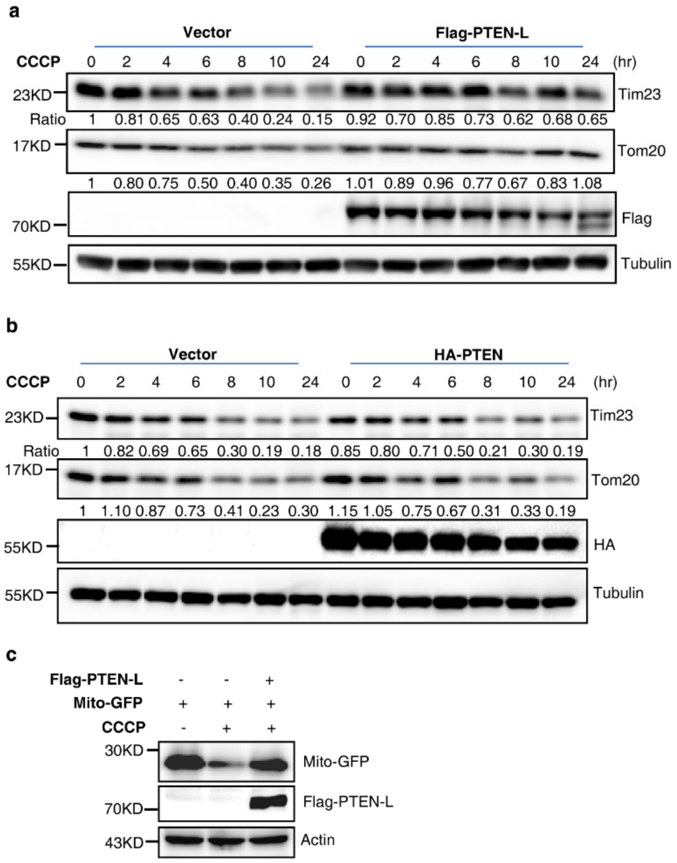


**Figure S2 PTEN-L, but not PTEN, inhibits mitophagy induced by CCCP. a** YFP-Parkin-HeLa cells were transiently transfected with plasmids encoding Flag-PTEN-L and treated with CCCP (20 µM) for indicated hours. Whole-cell lysates were analyzed by immunoblotting as indicated. The ratios of the relative band intensity were shown below the respective bands. **b** YFP-Parkin-HeLa cells were transiently transfected with plasmids encoding HA-PTEN and treated with CCCP (20 µM) for indicated hours. Whole-cell lysates were analyzed by immunoblotting as indicated. The ratios of the relative band intensity were shown below the respective bands. **c** YFP-Parkin-HeLa cells were transiently co-transfected with mitochondrial-targeted GFP plasmids and Flag-PTEN-L. Cells were treated with CCCP (20 µM) for 6 h and the whole-cell lysates were analyzed by immunoblotting as indicated.
