# Supplementary material for: PTEN-L is a novel protein phosphatase for ubiquitin dephosphorylation to inhibit PINK1–Parkin-mediated mitophagy
Source: Cell Res. 2018 Jun 22;28(8):787–802. doi: 10.1038/s41422-018-0056-0 (PMC6082900; doi:10.1038/s41422-018-0056-0)
Supplement: Supplementary file 3 — Supplementary information, Figure S3 [file 41422_2018_56_MOESM3_ESM.pdf]

Supplementary information, Figure S3

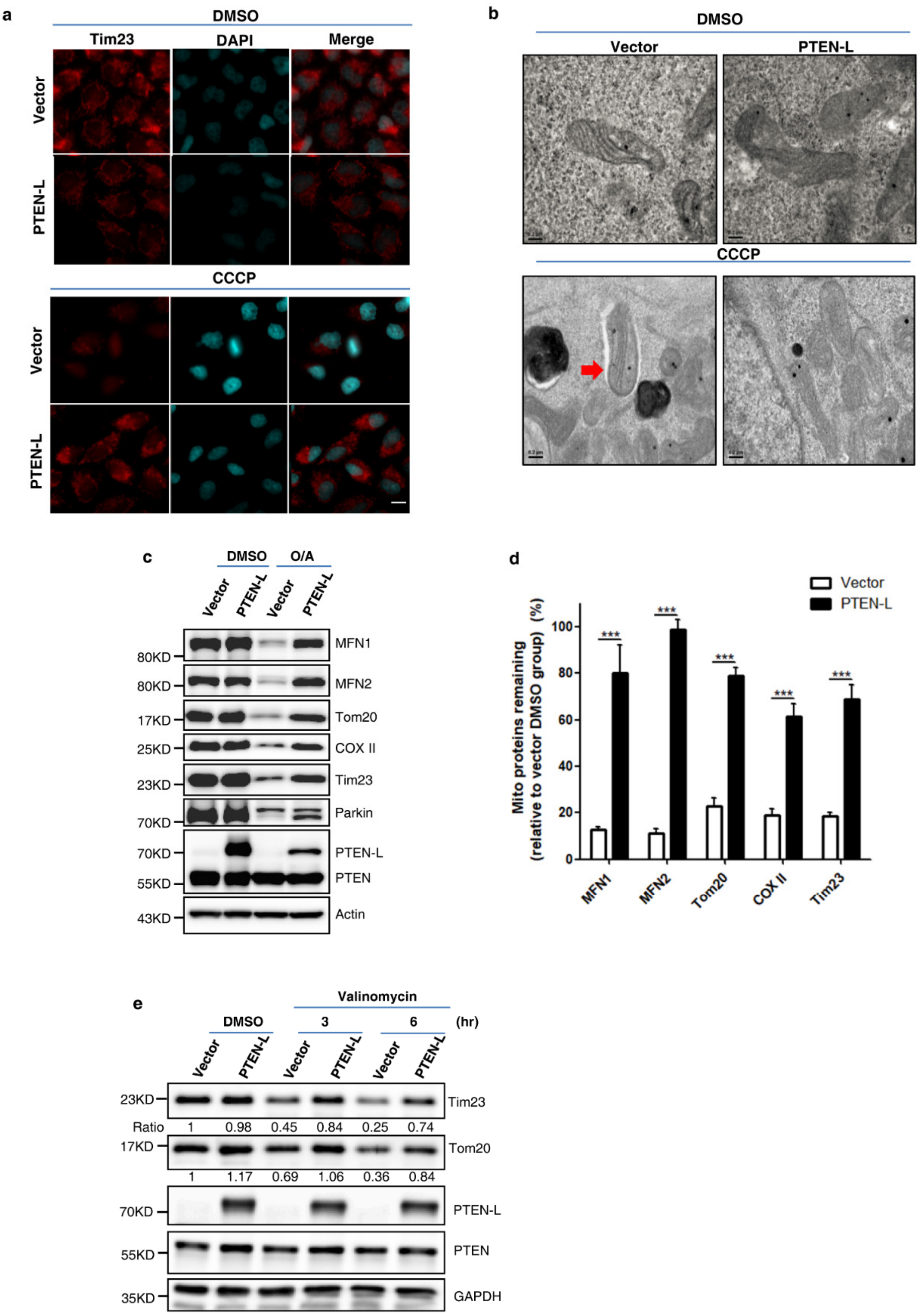

Figure S3 PTEN-L inhibits mitophagy induced by various mitochondria-damaging agents. **a** YFP-Parkin-HeLa cells with PTEN-L stable expression were treated with DMSO or CCCP (5 μM) for 24 h. Cells were subjected to immunostaining with Tim23 (Red) and Nucleus (DAPI, Cyan) and observed by fluorescent microscopy. Scale bar, 10 μm. **b** Transmission electron microscopy analysis of mitophagy in YFP-Parkin-HeLa cells with PTEN-L stable expression were treated with DMSO or CCCP (5 μM) for 6 h. Arrow indicates engulfed mitochondria in autophagosome. Scale bar, 0.2 μm. **c** YFP-Parkin-HeLa cells with PTEN-L stable expression or control vector were treated with O/A (10 nM and 100 nM) for 24 h and immunoblotting for mitochondrial proteins was performed as indicated. **d** Mitochondrial proteins from **c** were quantified and data is presented as mean ± SD from 3 independent experiments. ***P < 0.001 (two-way ANOVA). **e** YFP-Parkin-HeLa cells with PTEN-L stable expression or control vector were treated with valinomycin (50 nM) for 3 h or 6 h, and immunoblotting was performed as indicated. The ratios of the relative band intensity were shown below the respective bands.
