# Supplementary material for: PTEN-L is a novel protein phosphatase for ubiquitin dephosphorylation to inhibit PINK1–Parkin-mediated mitophagy
Source: Cell Res. 2018 Jun 22;28(8):787–802. doi: 10.1038/s41422-018-0056-0 (PMC6082900; doi:10.1038/s41422-018-0056-0)
Supplement: Supplementary file 4 — Supplementary information, Figure S4 [file 41422_2018_56_MOESM4_ESM.pdf]

Supplementary information, Figure S4

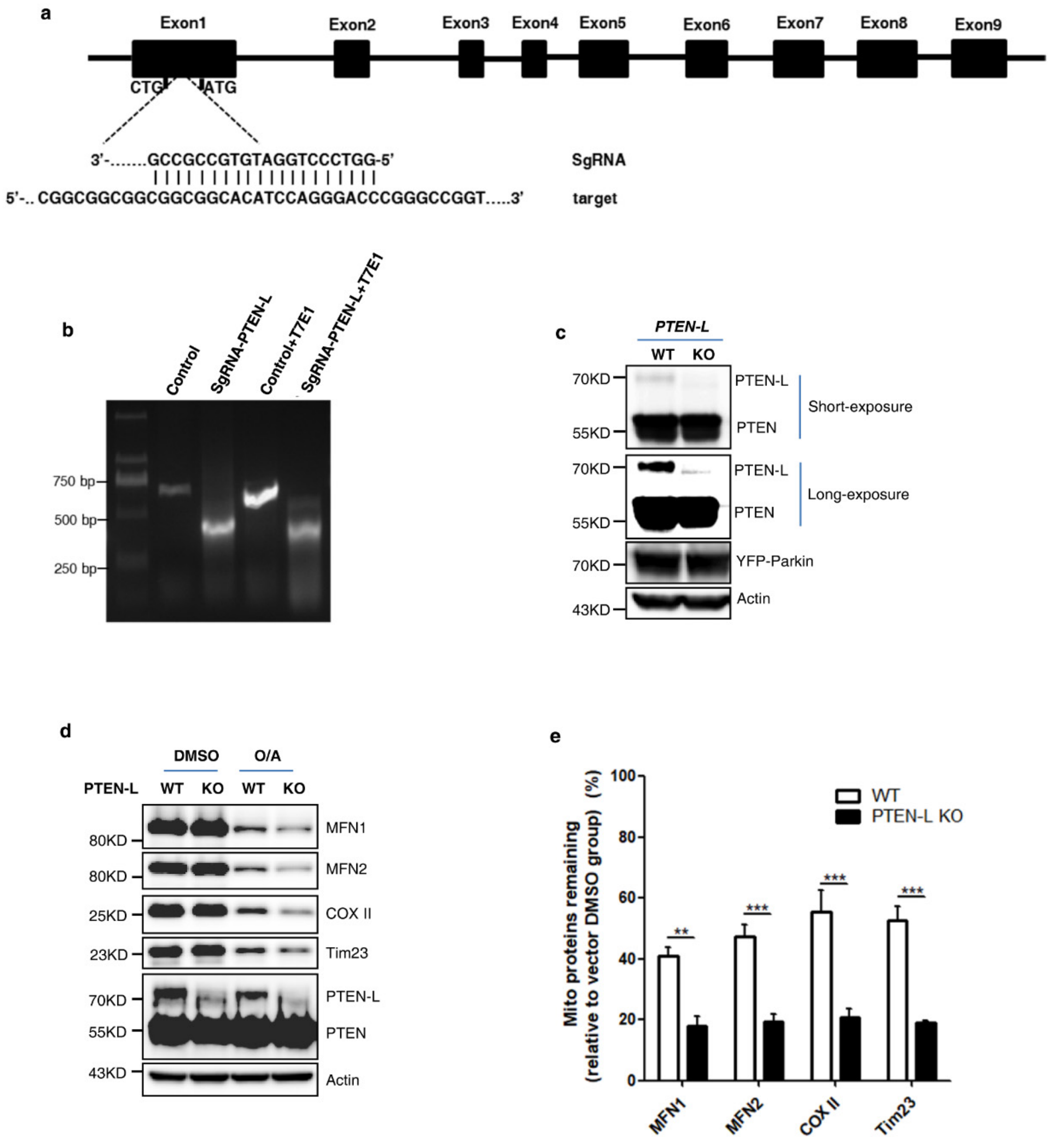

Figure S4 Knockout of *PTEN-L* with CRISPR/Cas9 technique sensitizes cells to mitophagy. a Schematic depiction of the *PTEN-L* locus. The sequence targeted by the small guide RNAs (sgRNA) is indicated by capital letters in the exon 1 of *PTEN-L.* Start codon CTG and ATG are highlighted in capital letters. **b** T7E1 assays of Cas9 mediated indels at the indicated targets in *PTEN-L* genome. PCR primers were designed in the upstream and downstream regions of the knockout site. The PCR products were annealed and digested with T7E1 and analyzed in agarose gel electrophoresis. **c** YFP-Parkin-HeLa cells with PTEN-L

knockout (PTEN-L KO) created via CRISPR/Cas9 were subjected to immunoblotting as indicated. **d** Wild-type (WT) and PTEN-L KO YFP-Parkin-HeLa cells were treated with O/A (4 nM and 40 nM) for 24 h and immunoblotting was performed as indicated. **e** Mitochondrial proteins from **d** were quantified and data is presented as mean ± SD from 3 independent experiments. $^{**}P < 0.01$, $^{***}P < 0.001$ (two-way ANOVA).
