# Supplementary material for: PTEN-L is a novel protein phosphatase for ubiquitin dephosphorylation to inhibit PINK1–Parkin-mediated mitophagy
Source: Cell Res. 2018 Jun 22;28(8):787–802. doi: 10.1038/s41422-018-0056-0 (PMC6082900; doi:10.1038/s41422-018-0056-0)
Supplement: Supplementary file 5 — Supplementary information, Figure S5 [file 41422_2018_56_MOESM5_ESM.pdf]

Supplementary information, Figure S5

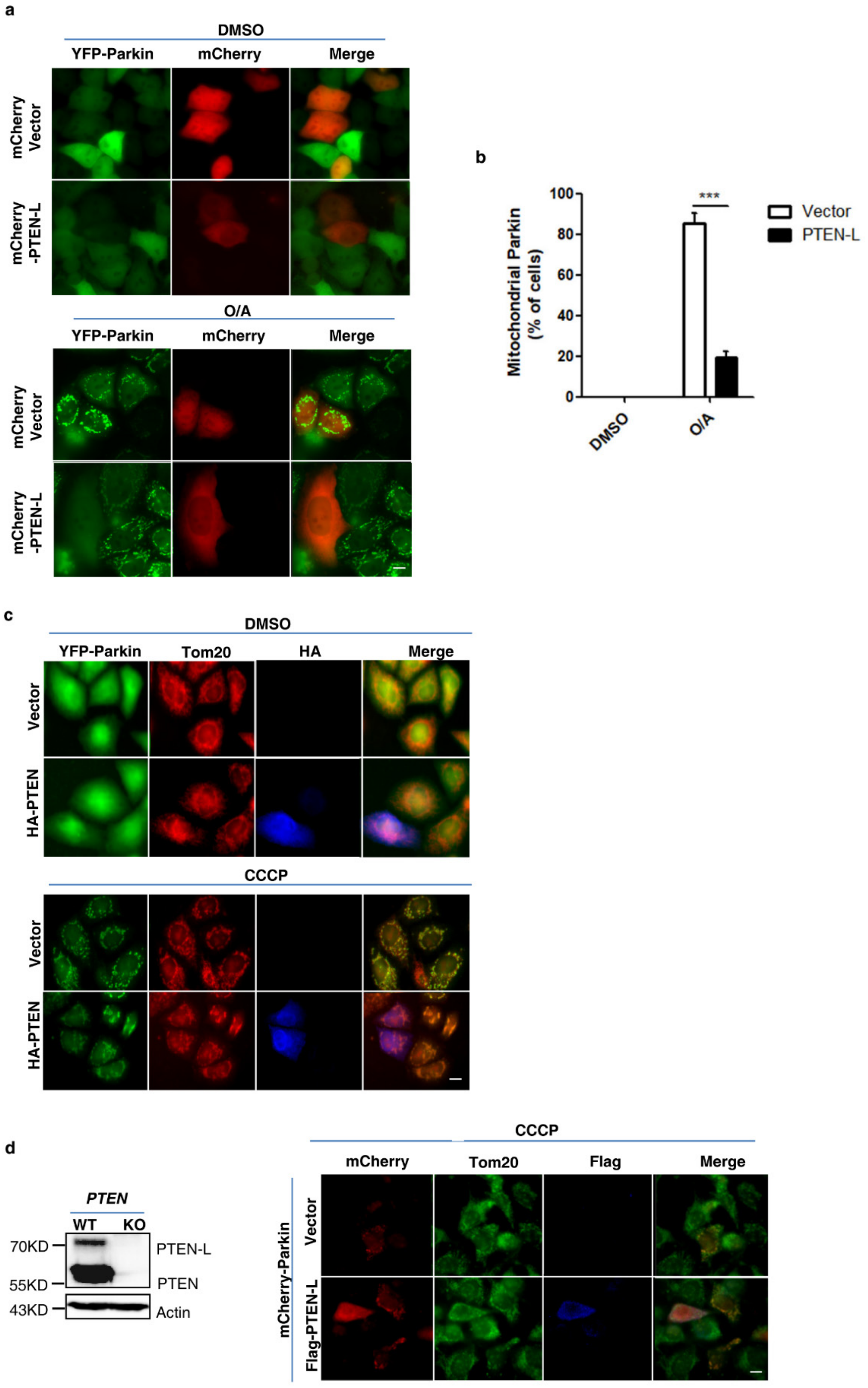

Figure S5 PTEN-L, but not PTEN, inhibits Parkin mitochondrial translocation induced by mitochondria-damaging agents. a YFP-Parkin-HeLa cells were transiently transfected with mCherry-PTEN-L and then treated with O/A (25 nM and 250 nM) for 2 h. YFP-Parkin (Green); mCherry (Red). Scale bar, 10 μm. **b** Percentage of cells with Parkin mitochondrial translocation was quantified by counting at least 300 cells after O/A treatment. Data is presented as mean ± SD from 3 independent experiments. ***$P < 0.001$ (two-way ANOVA). **c** YFP-Parkin-HeLa cells were transiently transfected with HA-PTEN plasmids and treated with DMSO or CCCP (5 μM) for 2 h. Parkin mitochondrial translocation and co-localization with mitochondrial protein Tom20 was observed by fluorescent microscopy. Tom20 (Red); YFP-Parkin (Green); HA-PTEN (Blue). Scale bar, 10 μm. **d** *PTEN*-knockout MEFs were confirmed by immunoblotting for both PTEN and PTEN-L (left panel) and were transiently transfected with plasmids encoding Flag-PTEN-L and mCherry-Parkin, and then treated with CCCP (10 μM) for 2 h (right panel). Mitochondrial translocation of mCherry-Parkin was analyzed by fluorescent microscopy. Tom20 (Green); mCherry-Parkin (Red); Flag-PTEN-L (Blue). Scale bar, 10 μm.
