# Supplementary material for: PTEN-L is a novel protein phosphatase for ubiquitin dephosphorylation to inhibit PINK1–Parkin-mediated mitophagy
Source: Cell Res. 2018 Jun 22;28(8):787–802. doi: 10.1038/s41422-018-0056-0 (PMC6082900; doi:10.1038/s41422-018-0056-0)
Supplement: Supplementary file 6 — Supplementary information, Figure S6 [file 41422_2018_56_MOESM6_ESM.pdf]

Supplementary information, Figure S6

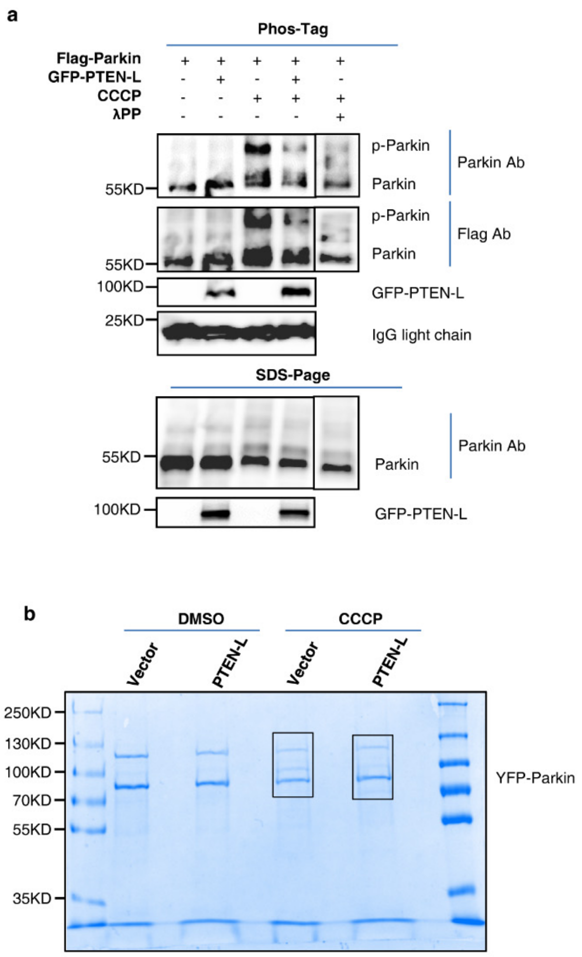

c

pSer65-Parkin

MATRIX SCIENCE Mascot Search Results

Peptide View

MS/MS Fragmentation of **NDWTVQNCDLDQQSIVHIVQRPWR**
Found in **tr|M4T4F2|M4T4F2_HUMAN** in **uni_human_i**, tr|M4T4F2|M4T4F2_HUMAN PARK2 splice variant OS=Homo sapiens GN=PARK2 PE=2 SV=1

Match to Query 3683: 3086.405772 from(1029.809200,3+) intensity(570384.3750) rtinseconds(2372) scans(13963) index(438)

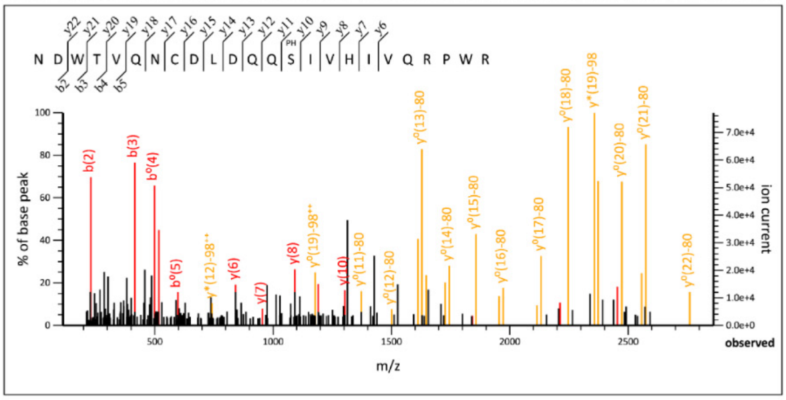

Monoisotopic mass of neutral peptide Mr(calc): 3086.4182
Fixed modifications: Carbamidomethyl (C) (apply to specified residues or termini only)
Variable modifications:
S14 : PhosphoST2 (ST), with neutral losses 97.9769(shown in table), 79.9663, 0.0000
Ions Score: 139 **Expect:** 1.6e-011
Matches : 49/530 fragment ions using 47 most intense peaks (help)

#	b	b^{++}	b^{*}	b^{*++}	b^{0}	b^{0++}	Seq.	y	y^{++}	y^{*}	y^{*++}	y^{0}	y^{0++}	#
1	115.0502	58.0287	98.0237	49.5155			N							24
2	**230.0771**	115.5422	213.0506	107.0289	212.0666	106.5369	D	2875.4057	1438.2065	2858.3791	1429.6932	2857.3951	1429.2012	23
3	**416.1565**	208.5819	399.1299	200.0686	398.1459	199.5766	W	***2760.3787***	1380.6930	2743.3522	1372.1797	2742.3681	1371.6877	22
4	**517.2041**	259.1057	500.1776	250.5924	499.1936	250.1004	T	***2574.2994***	1287.6533	2557.2729	1279.1401	2556.2888	1278.6481	21
5	616.2726	308.6399	599.2460	300.1266	598.2620	299.6346	V	***2473.2517***	1237.1295	2456.2252	1228.6162	2455.2412	1228.1242	20
6	744.3311	372.6692	727.3046	364.1559	726.3206	363.6639	Q	***2374.1833***	1187.5953	2357.1568	1179.0820	2356.1727	1178.5900	19
7	858.3741	429.6907	841.3475	421.1774	840.3635	420.6854	N	***2246.1247***	1123.5660	2229.0982	1115.0527	2228.1142	1114.5607	18
8	1018.4047	509.7060	1001.3782	501.1927	1000.3941	500.7007	C	***2132.0818***	1066.5445	2115.0553	1058.0313	2114.0712	1057.5393	17
9	1133.4316	567.2195	1116.4051	558.7062	1115.4211	558.2142	D	***1972.0512***	986.5292	1955.0246	978.0159	1954.0406	977.5239	16
10	1246.5157	623.7615	1229.4892	615.2482	1228.5051	614.7562	L	***1857.0242***	929.0157	1839.9977	920.5025	1839.0136	920.0105	15
11	1361.5427	681.2750	1344.5161	672.7617	1343.5321	672.2697	D	***1743.9401***	872.4737	1726.9136	863.9604	1725.9296	863.4684	14
12	1489.6012	745.3043	1472.5747	736.7910	1471.5907	736.2990	Q	***1628.9132***	814.9602	1611.8867	806.4470	1610.9026	805.9550	13
13	1617.6598	809.3335	1600.6333	800.8203	1599.6492	800.3283	Q	***1500.8546***	750.9310	1483.8281	742.4177	1482.8441	741.9257	12
14	1686.6813	843.8443	1669.6547	835.3310	1668.6707	834.8390	S	***1372.7960***	686.9017	1355.7695	678.3884	1354.7855	677.8964	11
15	1799.7653	900.3863	1782.7388	891.8730	1781.7548	891.3810	I	***1303.7746***	652.3909	1286.7480	643.8777			10
16	1898.8338	949.9205	1881.8072	941.4072	1880.8232	940.9152	V	***1190.6905***	595.8489	1173.6640	587.3356			9
17	2035.8927	1018.4500	2018.8661	1009.9367	2017.8821	1009.4447	H	***1091.6221***	546.3147	1074.5956	537.8014			8
18	2148.9767	1074.9920	2131.9502	1066.4787	2130.9662	1065.9867	I	***954.5632***	477.7852	937.5366	469.2720			7
19	2248.0451	1124.5262	2231.0186	1116.0129	2230.0346	1115.5209	V	***841.4791***	421.2432	824.4526	412.7299			6
20	2376.1037	1188.5555	2359.0772	1180.0422	2358.0932	1179.5502	Q	***742.4107***	371.7090	725.3842	363.1957			5
21	2532.2048	1266.6061	2515.1783	1258.0928	2514.1943	1257.6008	R	614.3521	307.6797	597.3256	299.1664			4
22	2629.2576	1315.1324	2612.2310	1306.6192	2611.2470	1306.1272	P	458.2510	229.6292	441.2245	221.1159			3
23	2815.3369	1408.1721	2798.3104	1399.6588	2797.3263	1399.1668	W	361.1983	181.1028	344.1717	172.5895			2
24							R	175.1190	88.0631	158.0924	79.5498			1

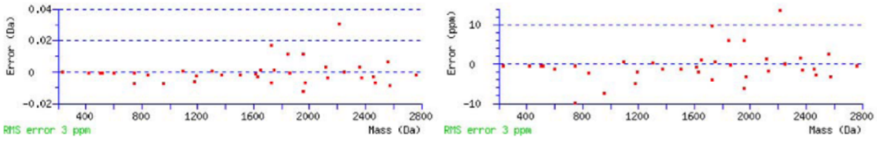

All matches to this query

Score	Mr(calc)	Delta	Sequence	Site Analysis
138.9	3086.4182	-0.0124	NDWTVQNCDLDQQSIVHIVQRPWR	PhosphoST2 S14 100.00%
38.0	3086.4182	-0.0124	NDWTVQNCDLDQQSIVHIVQRPWR	PhosphoST2 T4 0.00%

Figure S6 PTEN-L reduces pSer65-Parkin. a HEK293T cells transfected with Flag-Parkin and GFP-PTEN-L were treated with CCCP (10 μM) for 4 h. Flag-Parkin was pulled down with Flag beads. Immunoprecipitants were subsequently separated on a Phos-tag gel, followed by immunoblotting as indicated. Phosphorylated bands of Parkin were confirmed by their disappearance with Lambda protein phosphatase (λPP) treatment. SDS-PAGE was performed concurrently. **b** YFP-Parkin-HeLa cells with PTEN-L stable expression or control vector were treated with CCCP (10 μM) for 4 h. YFP-Parkin was pulled down by GFP-beads and subjected to SDS-PAGE and InstantBlue staining. Bound proteins in black boxes were excised for mass spectrometry analysis. **c** Mascot information with detailed b- and y-ion information of **NDWTVQNCDLDQQS(P)IVHIVQRPWR** of Parkin.
