# Supplementary material for: PTEN-L is a novel protein phosphatase for ubiquitin dephosphorylation to inhibit PINK1–Parkin-mediated mitophagy
Source: Cell Res. 2018 Jun 22;28(8):787–802. doi: 10.1038/s41422-018-0056-0 (PMC6082900; doi:10.1038/s41422-018-0056-0)
Supplement: Supplementary file 7 — Supplementary information, Figure S7 [file 41422_2018_56_MOESM7_ESM.pdf]

Supplementary information, Figure S7

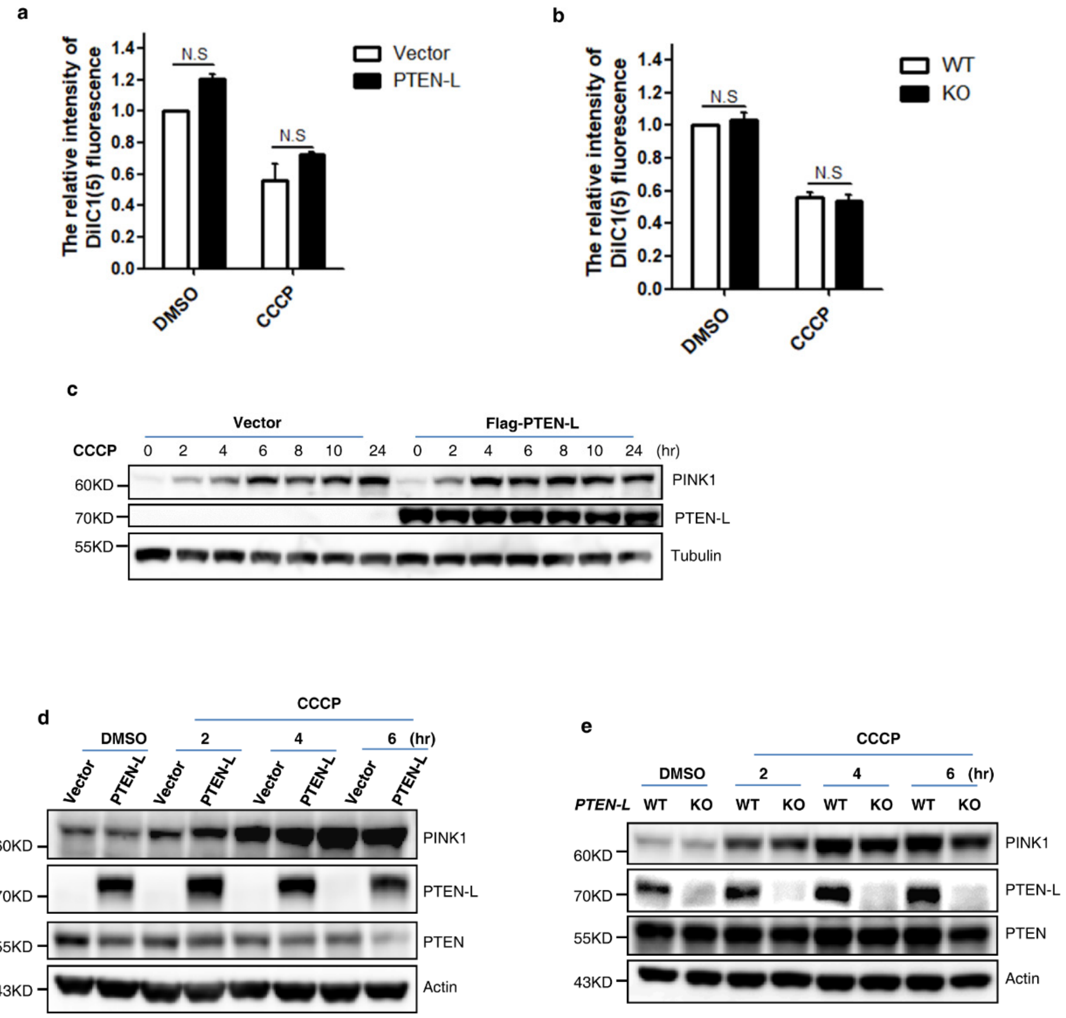

Figure S7 PTEN-L does not inhibit mitochondrial depolarization or PINK1 stabilization. a YFP-Parkin-HeLa cells with PTEN-L stable expression were treated with CCCP (50 μM) for 5 min. Cells were stained with DiIC1(5) (50 nM) for 20 min and a total of 10,000 cells were collected and quantified with a flow cytometer. Data is presented as mean ± SD from 3 independent experiments. N.S, no significant difference, $P > 0.05$ (two-way ANOVA). **b** WT and PTEN-L KO YFP-Parkin-HeLa cells were treated as in **a**. Data is presented as mean ± SD from 3 independent experiments. N.S, no significant difference, $P >$ 0.05 (two-way ANOVA). **c** YFP-Parkin-HeLa cells were transiently transfected with

plasmids encoding Flag-PTEN-L and treated with CCCP (20 μM) for indicated hours. Whole cell lysates were analyzed by immunoblotting. **d** YFP-Parkin-HeLa cells with PTEN-L stable expression or control vector were treated with CCCP (20 μM) for indicated hours and the whole cell lysates were analyzed by immunoblotting. **e** WT and PTEN-L KO YFP-Parkin-HeLa cells were treated with CCCP (20 μM) for indicated hours and the whole-cell lysates were analyzed by immunoblotting.
