# Supplementary material for: PTEN-L is a novel protein phosphatase for ubiquitin dephosphorylation to inhibit PINK1–Parkin-mediated mitophagy
Source: Cell Res. 2018 Jun 22;28(8):787–802. doi: 10.1038/s41422-018-0056-0 (PMC6082900; doi:10.1038/s41422-018-0056-0)
Supplement: Supplementary file 8 — Supplementary information, Figure S8 [file 41422_2018_56_MOESM8_ESM.pdf]

Supplementary information, Figure S8

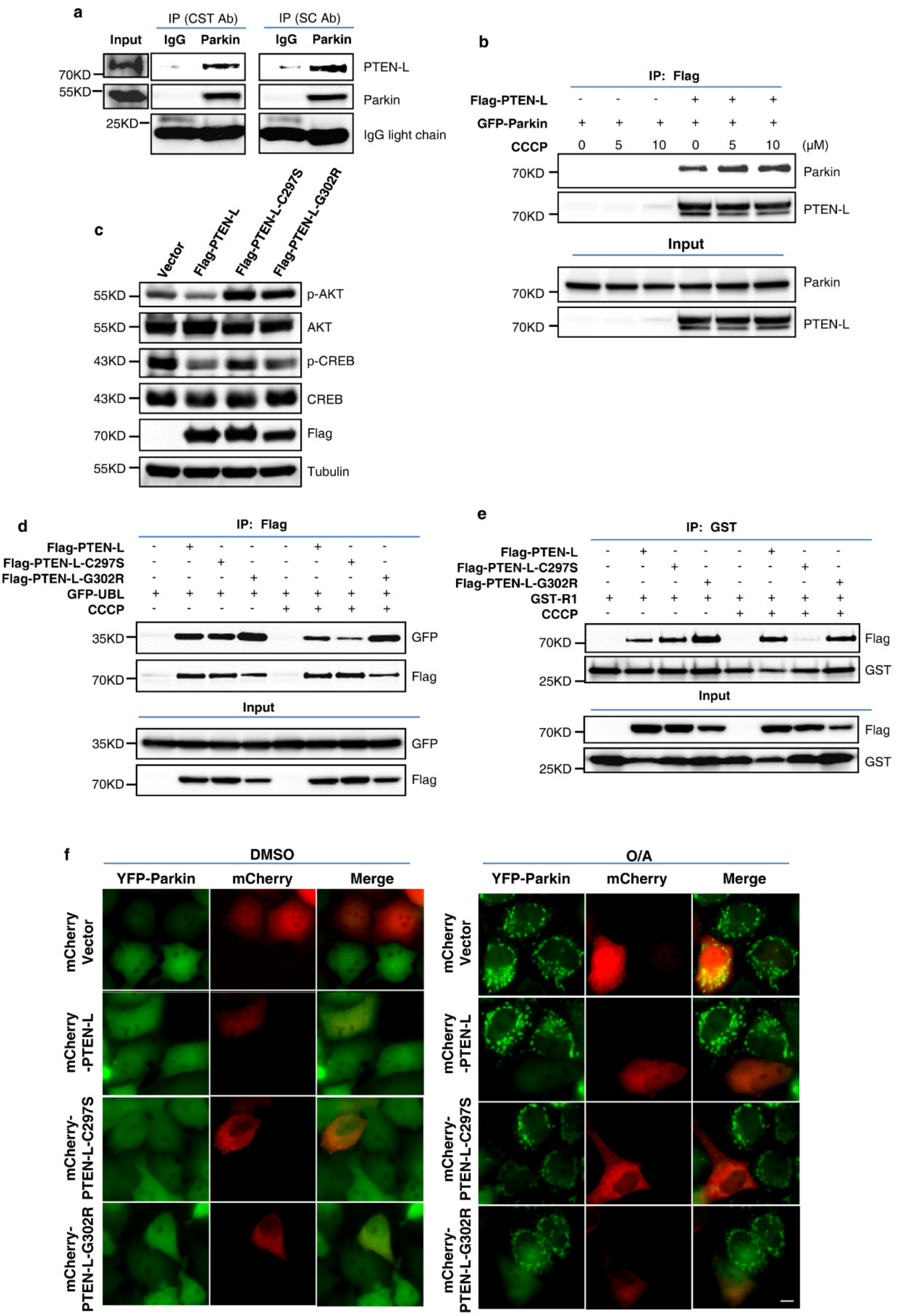

Figure S8 PTEN-L interacts with Parkin and blocks Parkin translocation in a protein phosphatase activity-dependent manner. a Parkin was immunoprecipitated with different anti-Parkin antibodies (CST, Cell Signaling Technology; SC, Santa Cruz) from mouse brain tissue homogenate followed by immunoblotting as indicated. **b** HEK293T cells were transiently co-transfected with GFP-Parkin and Flag-PTEN-L plasmids. Cells were treated with the indicated concentrations of CCCP for 2 h. PTEN-L was immunoprecipitated (IP) with anti-Flag antibody followed by immunoblotting for Parkin. **c** HEK293T cells were transiently transfected with PTEN-L, PTEN-L-C297S or PTEN-L-G302R. Whole-cell lysates were analyzed by immunoblotting as indicated. **d** HEK293T cells were transiently co-transfected with Flag-tagged PTEN-L or its two phosphatase mutants and Parkin truncation mutant GFP-UBL. Cells were treated with or without CCCP (5 μM) for 4 h. PTEN-L and its mutants were immunoprecipitated (IP) with anti-Flag antibody followed by immunoblotting as indicated. **e** HEK293T cells were transiently co-transfected with Flag-tagged PTEN-L or its two phosphatase mutants and Parkin truncation mutant GST-RING1 (R1). Cells were treated with or without CCCP (5 μM) for 4 h. RING1 was immunoprecipitated (IP) with anti-GST antibody followed by immunoblotting with the indicated antibodies. **f** YFP-Parkin-HeLa cells were transiently transfected with mCherry-tagged PTEN-L or its two phosphatase mutants. Cells were treated with O/A (25 nM and 250 nM) for 2 h. Mitochondrial translocation of YFP-Parkin was analyzed by fluorescent microscopy. YFP-Parkin (Green), mCherry (Red). Scale bar, 10 μm.
