# Supplementary material for: PTEN-L is a novel protein phosphatase for ubiquitin dephosphorylation to inhibit PINK1–Parkin-mediated mitophagy
Source: Cell Res. 2018 Jun 22;28(8):787–802. doi: 10.1038/s41422-018-0056-0 (PMC6082900; doi:10.1038/s41422-018-0056-0)
Supplement: Supplementary file 9 — Supplementary information, Figure S9 [file 41422_2018_56_MOESM9_ESM.pdf]

Supplementary information, Figure S9

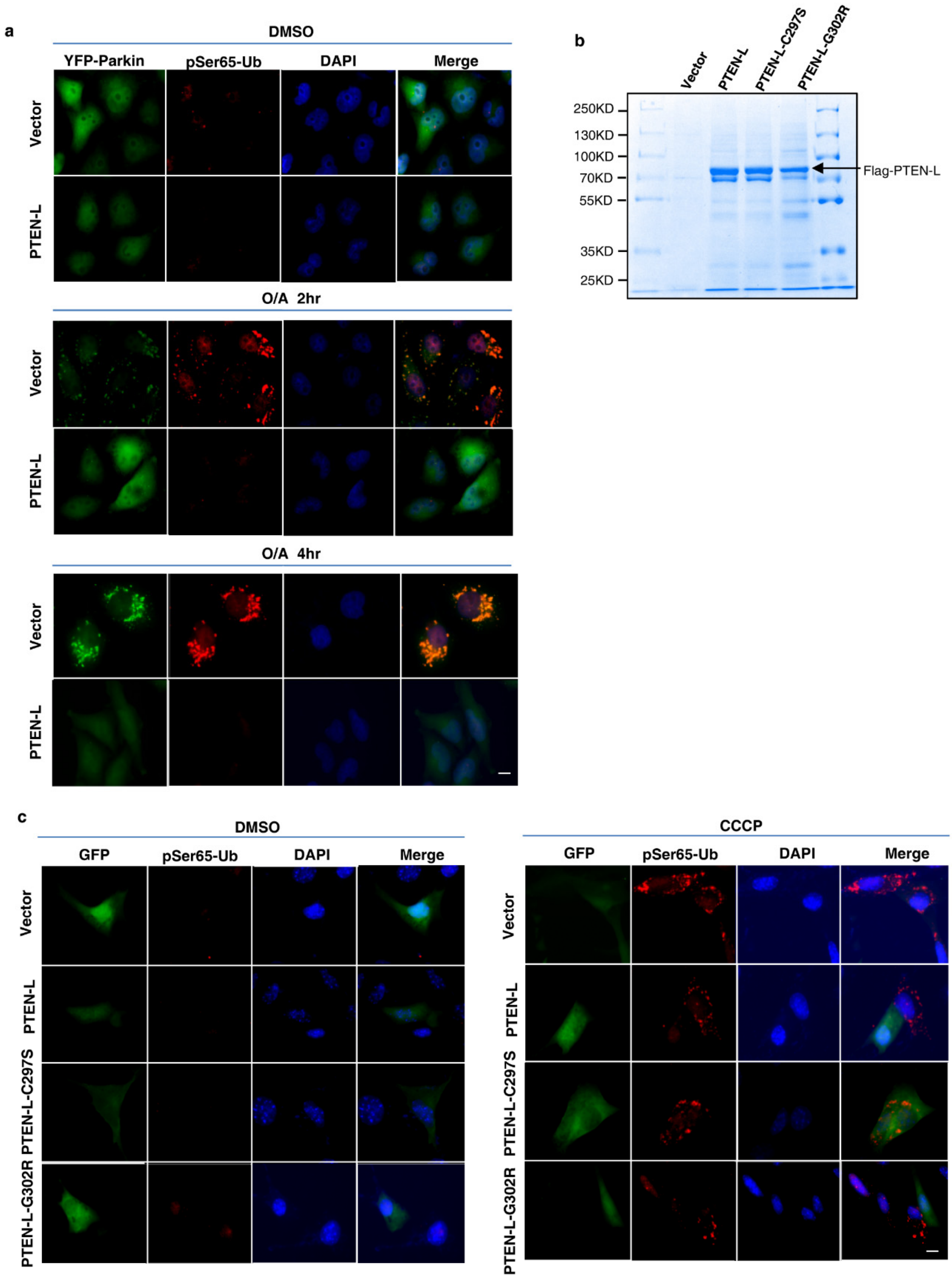

Figure S9 PTEN-L dephosphorylates ubiquitin in a protein phosphatase-dependent manner. **a** YFP-Parkin-HeLa cells with PTEN-L stable expression or control vector were treated with O/A (25 nM and 250 nM) for indicated hours. pSer65-Ub was immunostained by anti-pSer65-Ub antibody and observed by fluorescent microscopy. pSer65-Ub (Red); YFP-Parkin (Green); Nucleus (DAPI, Blue). Scale bar, 10 μm. **b** HEK293T cells were transiently transfected with Flag-tagged PTEN-L or its two phosphatase mutants. PTEN-L and its mutants were pulled down by Flag beads, eluted by Flag peptide, and subjected to SDS-PAGE and InstantBlue staining. **c** MEFs stably expressing HA-Parkin were transiently transfected with plasmids encoding GFP-PTEN-L and the two GFP-PTEN-L mutants, and then treated with CCCP (5 μM) for 3 h. pSer65-Ub was immunostained by anti-pSer65-Ub antibody and observed by fluorescent microscopy. pSer65-Ub (Red); GFP-PTEN-L and mutants (Green); Nucleus (DAPI, Blue). Scale bar, 10 μm.
