# Supplementary material for: PTEN-L is a novel protein phosphatase for ubiquitin dephosphorylation to inhibit PINK1–Parkin-mediated mitophagy
Source: Cell Res. 2018 Jun 22;28(8):787–802. doi: 10.1038/s41422-018-0056-0 (PMC6082900; doi:10.1038/s41422-018-0056-0)
Supplement: Supplementary file 10 — Supplementary information, Figure S10 [file 41422_2018_56_MOESM10_ESM.pdf]

Supplementary information, Figure S10

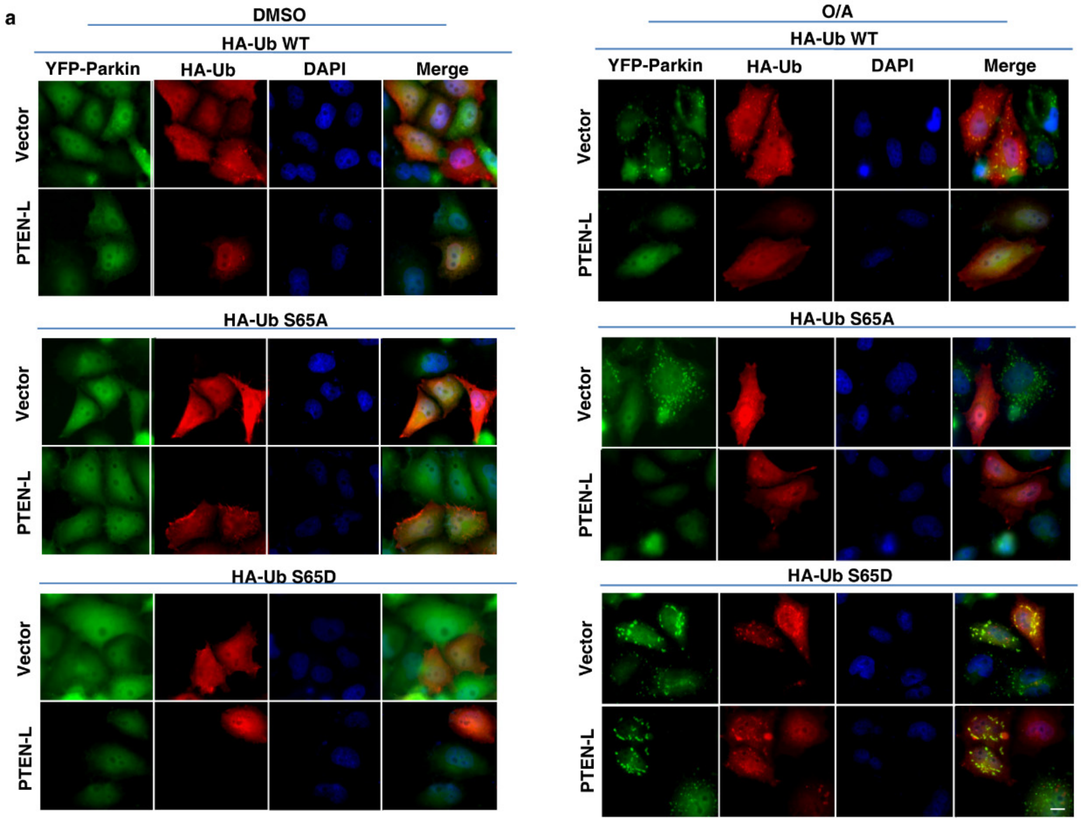

b **p-Ser65-Ub**

Mascot Search Results

Peptide View

MS/MS Fragmentation of **TLSDYNIQKESTLHLVLR**
Found in **sp|P62987|RL40_HUMAN** in **uni_human_nr**, Ubiquitin-60S ribosomal protein L40 OS=Homo sapiens GN=UBA52 PE=1 SV=2

Match to Query 252304: 2209.113628 from(1105.564090,2+) intensity(3893247.7500) rtinseconds(1972) scans(11546) index(100927)

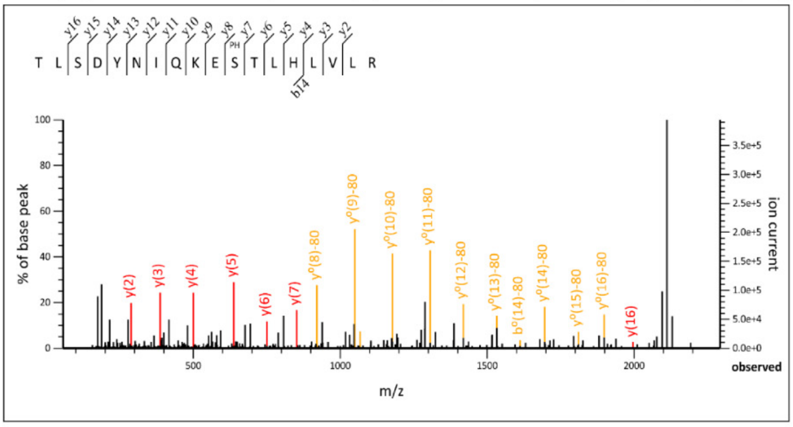

Monoisotopic mass of neutral peptide Mr(calc): 2209.1144
Fixed modifications: Carbamidomethyl (C) (apply to specified residues or termini only)
Variable modifications:
S11 : PhosphoST2 (ST), with neutral losses 97.9769(shown in table), 79.9663, 0.0000
Ions Score: 147 **Expect:** 1.4e-012
Matches : 28/386 fragment ions using 21 most intense peaks (help)

#	b	b^{++}	b*	b^{*++}	b^0	b^{0++}	Seq.	y	y^{++}	y*	y^{*++}	y^0	y^{0++}	#
1	102.0550	51.5311			84.0444	42.5258	T							18
2	215.1390	108.0731			197.1285	99.0679	L	2011.0971	1006.0522	1994.0706	997.5389	1993.0865	997.0469	17
3	302.1710	151.5892			284.1605	142.5839	S	*1898.0130*	949.5102	1880.9865	940.9969	1880.0025	940.5049	16
4	417.1980	209.1026			399.1874	200.0974	D	*1810.9810*	905.9941	1793.9545	897.4809	1792.9704	896.9889	15
5	580.2613	290.6343			562.2508	281.6290	Y	*1695.9541*	848.4807	1678.9275	839.9674	1677.9435	839.4754	14
6	694.3042	347.6558	677.2777	339.1425	676.2937	338.6505	N	*1532.8907*	766.9490	1515.8642	758.4357	1514.8802	757.9437	13
7	807.3883	404.1978	790.3618	395.6845	789.3777	395.1925	I	*1418.8478*	709.9275	1401.8213	701.4143	1400.8372	700.9223	12
8	935.4469	468.2271	918.4203	459.7138	917.4363	459.2218	Q	*1305.7637*	653.3855	1288.7372	644.8722	1287.7532	644.3802	11
9	1063.5419	532.2746	1046.5153	523.7613	1045.5313	523.2693	K	*1177.7052*	589.3562	1160.6786	580.8429	1159.6946	580.3509	10
10	1192.5844	596.7959	1175.5579	588.2826	1174.5739	587.7906	E	*1049.6102*	525.3087	1032.5837	516.7955	1031.5996	516.3035	9
11	1261.6059	631.3066	1244.5794	622.7933	1243.5953	622.3013	S	*920.5676*	460.7874	903.5411	452.2742	902.5570	451.7822	8
12	1362.6536	681.8304	1345.6270	673.3172	1344.6430	672.8251	T	*851.5461*	426.2767	834.5196	417.7634	833.5356	417.2714	7
13	1475.7377	738.3725	1458.7111	729.8592	1457.7271	729.3672	L	*750.4985*	375.7529	733.4719	367.2396			6
14	**1612.7966**	806.9019	1595.7700	798.3886	1594.7860	797.8966	H	*637.4144*	319.2108	620.3879	310.6976			5
15	1725.8806	863.4440	1708.8541	854.9307	1707.8701	854.4387	L	*500.3555*	250.6814	483.3289	242.1681			4
16	1824.9490	912.9782	1807.9225	904.4649	1806.9385	903.9729	V	*387.2714*	194.1394	370.2449	185.6261			3
17	1938.0331	969.5202	1921.0066	961.0069	1920.0225	960.5149	L	*288.2030*	144.6051	271.1765	136.0919			2
18							R	175.1190	88.0631	158.0924	79.5498			1

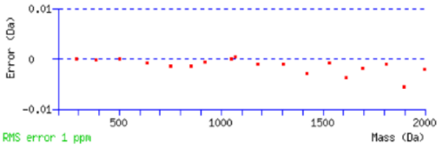

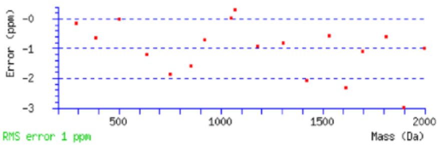

All matches to this query

Score	Mr(calc)	Delta	Sequence	Site Analysis
146.8	2209.1144	-0.0007	TLSDYNIQKESTLHLVLR	PhosphoST2 S11 98.22%
129.4	2209.1144	-0.0007	TLSDYNIQKESTLHLVLR	PhosphoST2 T12 1.78%
86.1	2209.1144	-0.0007	TLSDYNIQKESTLHLVLR	PhosphoST2 S3 0.00%
69.2	2209.1144	-0.0007	TLSDYNIQKESTLHLVLR	PhosphoST2 T1 0.00%
8.1	2209.0966	0.0170	SSAPMGIYQKQNLTKIPPR	
0.4	2209.0966	0.0170	SSAPMGIYQKQNLTKIPPR	

Figure S10 The role of ubiquitin phosphorylation status in PTEN-L's effect on Parkin translocation. a YFP-Parkin-HeLa cells with PTEN-L stable expression or control vector were transiently transfected with plasmids encoding HA-Ub WT or two HA-Ub mutants (S65A and S65D) and treated with DMSO or O/A (25 nM and 250 nM) for 2 h. Parkin mitochondrial translocation was observed by fluorescent microscopy. YFP-Parkin (Green); HA-Ub (Red); Nucleus (DAPI, Blue). Scale bar, 10 μm. **b** Mascot information with detailed b- and y-ion information of **TLSDYNIQKESTLHLVLR** of ubiquitin.
