# Supplementary material for: PTEN-L is a novel protein phosphatase for ubiquitin dephosphorylation to inhibit PINK1–Parkin-mediated mitophagy
Source: Cell Res. 2018 Jun 22;28(8):787–802. doi: 10.1038/s41422-018-0056-0 (PMC6082900; doi:10.1038/s41422-018-0056-0)
Supplement: Supplementary file 13 — Supplementary movie legend [file 41422_2018_56_MOESM13_ESM.docx]

Supplementary information, Movie S1

YFP-Parkin-HeLa cells were transiently transfected with mCherry-PTEN-L and treated with CCCP (5 µM) for 2 h. Imaging was started immediately after adding CCCP. YFP-Parkin (Green); mCherry-PTEN-L (Red). Scale bar, 50 µm.

Supplementary information, Movie S2

YFP-Parkin-HeLa without (WT) or with PTEN-L KO (KO) cells were treated with CCCP (4 µM) for 4 h. Imaging was started immediately after adding CCCP. YFP-Parkin (Green). Scale bar, 50 µm.
